# New extension of ordinal priority approach for multiple attribute decision-making problems: design and analysis

**DOI:** 10.1007/s40747-022-00721-w

**Published:** 2022-04-29

**Authors:** Mohamed Abdel-Basset, Mai Mohamed, Ahmed Abdel-monem, Mohamed Abd Elfattah

**Affiliations:** 1 Faculty of Computers and Informatics, Zagazig University, Zagazig, 44519 Sharqiyah Egypt; 2 Misr Higher Institute for Commerce and Computers, Mansoura, Egypt

**Keywords:** Robot selection, Ordinal priority approach (OPA), Neutrosophic sets, Triangular neutrosophic numbers, Uncertainty, Multi-attribute decision-making

## Abstract

The selection and assessment process of appropriate robots became a more complex and complicated task due to various available alternatives and conflicting attributes which must take into consideration. Also, uncertainty which exists usually in the selection process is an unavoidable component that needs to be thoughtfully measured and traditional multi-attribute decision-making approaches failed to deal precisely with it. Since almost all decisions originate from subjective ordinal preferences, handling uncertainty using linguistic variables is also not enough. Thus, the objective of the current study is to present a new extended ordinal priority approach in the neutrosophic environment for the first time to select an appropriate robot. Since neutrosophic is one of the most effective and accommodating tools for handling uncertainty, thus, this method goes to transform linguistic information into triangular neutrosophic numbers using a new presented scale. This scale was used to determine the importance degree of attributes and alternatives regarding experts' opinions. Also, the score function of the triangular neutrosophic number is used for prioritizing attributes and alternatives. The experts in our proposed method have the same degree of importance, since each expert is a person with special skills and knowledge representing mastery of a particular subject. To measure the applicability and efficiency of the proposed approach, an experimental case study has been established for the robot selection problem of a new pharmaceutical city in Egypt for the first time. The source of data in this case study is experts, interviews, and questionnaires. Also, sensitivity and comparative analysis are further made for verifying the power of the proposed approach. The outcome of this study shows that the suggested approach for robot selection is quite helpful and has a great performance under uncertainty over classical and fuzzy ordinal priority approaches. Also, the suggested approach is less consumption of time and simpler than the fuzzy ordinal priority approach. Therefore, we recommend firms and governments to apply it for increasing product quality, hence the profitability of manufacturing industries and decrease needless costs.

**Supplementary Information:**

The online version contains supplementary material available at 10.1007/s40747-022-00721-w.

## Introduction

As we know a robot is a power-driven self-controlled programmable machine. It is made with mechanical, microelectronic, and electrical components. Robots can frequently perform complex and repetitive tasks. It also can be considered as a multi-functional structure, that can be well controlled by programs and commands [1]. The use of robots in commercial ventures and production units has been extended in the last decades to utilize resources well in time to enhance the efficiency and quality of products.

The robotic system can be used in many systems like auto manufacturing, firm manufacturing in the welding sector, large pharmaceutical companies, etc. As robots are very expensive, then a detailed study for selecting the appropriate one for using it in the appropriate location must be made.

The selection process of an appropriate robot depends on multiple attributes that affect this selection such as accuracy, safety, security, performance, and speed or velocity of robot. This study takes into consideration the swarm intelligence, IoT, and big data attributes. We considered these attributes in the evaluation process, because they add value to the robot selection problem. For example, the robot should have big data processing that means capability and memory capacity for storing objects, maps, and images. Also, the cloud computing attribute is essential in this study, because robots can connect to cities in Europe and America to transfer many skills in the production of pharmaceutical. Selecting it also based on IoT means using sensors that are connected to others for doing different tasks as vehicles sensors which are used for vehicles’ detection and measuring the amount of wetness of the road [2].

With these enormous attributes, it is not easy to select a suitable robot by decision-makers from among available robots in the marketplace. Therefore, decision-makers needs to use a suitable model for the best selection of robots and then achieve the desired task.

Since the selection process of the appropriate robot is a multi-attribute decision-making (MADM) problem, several MADM techniques were applied to select the appropriate one [3]. However, there does not exist any research until now that used OPA for selecting appropriate robot. The OPA technique has several advantages such as (1) never needing a pairwise comparison matrix, (2) solving MADM problem using a mathematical model, (3) it also does not need normalization, and finally, it is simple and easy to understand. However, classical MADM techniques failed to handle uncertainty which exist usually in reality since in a classical set the element either belongs to a set or is excluded from it. Also, the fuzzy set considers only truth-membership degrees and is unable to handle indeterminacy degrees. Using a neutrosophic set in MADM make the decision-making process simulate reality via considering all aspect of decision (i.e., agree or truth, not sure or indeterminacy, and disagree or falsity degrees). Not only this, the neutrosophic set can handle paraconsistent information in contrast to the classical and fuzzy set. Also, in neutrosophic set, indeterminacy degree does not depend on truth and falsity degree and this can deal with various existing states. Also, using neutrosophic, we can distinguish between relative truth and absolute truth, and similarly relative falsehood and absolute falsehood. Due to this important role of the neutrosophic set, several researchers applied it in MADM as in [4]. However, in this study, we are the first to use the ordinal priority approach (OPA) in the neutrosophic environment for selecting the appropriate robot for the new pharmaceutical city in Egypt. The proposed method exemplifies an intellectual MADM methodology that can handle the linguistic variables represented by neutrosophic numbers with many conveniences. Also, it is simple and with a lower computational cost. It can also deal with indeterminacy and simulate a natural decision-making process. Also, the proposed method is less consuming of time than fuzzy OPA. Table 1 illustrates a comparison of the suggested approach with some other multi-attribute decision-making techniques.

**Table 1:** Comparison of the suggested approach with some other MADM approaches

	AHP	VIKOR	COPRAS	TOPSIS	PROMETHEE	BWM	ANP	OPA	OPA_F	OPA_N
Require a pairwise comparison?	Yes	No	No	No	Yes	Yes	Yes	No	No	No
Require a decision-making matrix?	No	Yes	Yes	Yes	Yes	No	No	No	No	Yes
Require converting qualitative variables into quantitative numbers?	Yes	Yes	Yes	Yes	Yes	Yes	Yes	No	Yes	Yes
Require another method for ranking alternatives?	No	No	No	No	No	No	No	No	No	No
Determine weights of attributes?	Yes	No	No	No	No	Yes	Yes	Yes	Yes	Yes
Construct a mathematical model?	No	No	No	No	No	Yes	No	Yes	Yes	Yes
Cost or benefits criteria can impact the process of decision-making?	No	Yes	Yes	Yes	Yes	No	No	No	No	No
Able to handle uncertainty?	No	No	No	No	No	No	No	No	In a small proportion	Yes
Able to consider Para consistency?	No	No	No	No	No	No	No	No	No	Yes
Simulate natural decision-making?	No	No	No	No	No	No	No	No	No	Yes

Besides key contributions of the suggested study, which was illustrated previously, we faced many challenges as follows: (1). The OPA method is new and there exist only a few research papers and never been presented in a neutrosophic environment. (2). In our study, we worked on a large scale of data. (3). In past research, there are a limited number of objective and subjective attributes for selecting an appropriate robot. However, in our study, we used 15 attributes and ten alternatives with five experts

The remaining parts of this research consist of the following: In Sect. 2, we presented the literature review. The algorithm of the classical OPA model is presented in Sect. 3. Section 4 discusses the fundamental steps of the neutrosophic ordinal priority approach (OPA-N). In Sect. 5, the actual case study for the selection of an appropriate robot is implemented. A sensitivity analysis is presented in Sect. 6. In Sect. 7, a comparative study of the proposed method with the other existing methods is presented in detail. The managerial implications of the proposed method are presented in Sect. 8. Section 9 presents the conclusion, findings, and offers future work suggestions.

## Literature review

The robot selection problem is solved by several MADM techniques, because it is a highly complex problem. Many past studies dealt with objective and subjective attributes. However, information son objective attributes is used more than subjective attributes.

Rashid et al. [5] used BW and EDAS methods for optimal industrial selection of robots. BW is used for calculating weights of the attribute, and EDAS used for ranking alternatives. They used four attributes, three objective attributes (Load Capacity, Repeatability, and Velocity Ratio), one subjective attribute (Degree of Freedom), and five alternatives with one expert. They concluded that their proposed model has advantages as fewer calculations and more consistency. The main limitations of their work are that they used small dimensions in calculations and failed to deal with vague, incomplete, and indeterminate information.

Also, Papakostas et al. [6] used the TOPSIS method for ranking fourteen alternatives with eight subjective attributes as (Multidimensional Learning, Flexibility, Cost, Resemblance of Human Abilities, Programmability, Autonomy, Hardware Performance, Factory Educational Abilities) for selecting appropriate social robot for education.

Xue et al. [7] made a case study for evaluating robots in a manufacturing firm. They used six attributes: three subjective attributes (Man-machine Interface, Flexibility of Programming, and Contract Vendor Service), three objective attributes (Purchase Cost, Capacity, Accuracy of Positioning), and three available alternatives (robots). They used the numbers of hesitant 2-tuple for evaluating attributes and alternatives with incomplete and vague information. They used an integrated MADM with an extended QUALIFLEX method for the selection process of the best robot. The main limitations of their work are that they did not consider indeterminacy degree; also, the number of attributes and alternatives is limited.

Nasrollahi et al. [8] proposed a fuzzy Best Worst Method and PROMETHEE for obtaining the weights of attributes and ranking the robots. They used six attributes with four alternatives which include four objectives like (Velocity, Cost, Repeatedly, and Capacity) and two subjective attributes (Flexibility of Programming and Man–Machine Interface). They used MATLAB software for ranking the robots. The main results of their work illustrated that their model is suitable and helpful for decision-makers in the selection process of appropriate robots. Also, they considered both quantitative and qualitative attributes. However, they failed to deal with uncertainty which exist usually in reality. They also did not compare their results with the other previous studies to show the difference between previous work methods and their method.

Rashid et al. [9] used the Best Worst Method, TOPSIS, and VIKOR methods integrated with Interval-Valued Trapezoidal fuzzy for selecting a suitable robot of a company. They used five robots and six attributes that include three subjective (Man–Machine Interface, Flexibility of Programming, and Vendor Contract of Vendor Service) and three objective attributes (Purchase Cost, Capacity of Load, and Accuracy of Positioning). The main outcome of their work is that they found the TOPSIS method which is more stable than the VIKOR method.

Kavita Devi [10] used the extension of the VIKOR method under an intuitionistic fuzzy environment for robot selection problems. He used three alternatives with three subjective (Man–Machine Interface, Programming Flexibility, and Vendor’s Service Contract) and three objective attributes (Purchase Cost, Load Capacity, and Positioning Accuracy).

Narayanamoorthy et al. [11] used Interval-valued intuitionistic hesitant fuzzy with entropy and VIKOR methods. The entropy method is used for calculating weights of attributes and the VIKOR method is used for rank alternatives. They used three alternatives, three subjective attributes (Programming, Performance, and Flexibility), and two objective attributes (Cost and Capacity).

Irfan Deli [12] used generalized trapezoidal hesitant fuzzy numbers with the TOPSIS method for the robot selection problem. He made his model for Auto Company. He used six subjective attributes (Inconsistency with infrastructure, Man-machine interface, Programming flexibility, Vendor’s service contract, Supporting channel partner’s performance, and Compliance) and five alternatives with one expert.

Also, Ghorabaee [13] used interval type-2 fuzzy sets with the VIKOR method for the robot selection problem. He used eight alternatives with seven subjective attributes (Inconsistency with infrastructure, Man-machine interface, Programming flexibility, Vendor's service contract, Supporting channel partner's performance, Compliance, and Stability) and one expert. He made his study for an auto company that wants to select an appropriate robot for the productions process.

Although several researchers used different MADM techniques for the robot selection process, they all failed to deal effectively with uncertainty which exist usually in reality. Also, the OPA technique was never used until now for robot selection problems and this motivated us to use it in this important problem.

Since OPA is a new technique, there exist few research papers until now which applied this technique. The ordinal priority approach (OPA) was proposed by Ataei et al. [14] to handle various drawbacks of traditional multi-attribute decision-making techniques. Also, a novel project portfolio selection framework toward organizational resilience based on robust OPA has been presented by Mahmoudi et al. [15]. A hybrid DEA-OPA Model has been presented also by Mahmoudi et al. [16] for evaluating the performance of the suppliers. Not only this, the OPA has been deployed by Mahmoudi and Javed [17] for evaluating the performance of construction subcontractors. The OPA was also used by Sadeghi et al. [18] for evaluating barriers to the sustainable construction industry.

Although the traditional OPA succeeded in handling the existing drawbacks of MADM, it failed to consider the situations in which experts are not sure about his/her opinion. To handle these drawbacks, a grey ordinal priority approach has been presented for selecting a sustainable supplier [19]. Since fuzzy sets are applied in various fields due to their important role [20], a fuzzy OPA has been presented also for selecting the resilient supplier and making a decision in the post-COVID era [21]. Not only this, the OPA was integrated with TOPSIS for large-scaled multiple attribute decision-making with missing values [22].

Due to the important role of the neutrosophic set in handling uncertainty via considering truth, indeterminacy, and falsity degrees [23–31], and hence simulating the natural decision-making process, we integrated it with OPA in this research for the first time to select appropriate robot for a new pharmaceutical city in Egypt. In this research, 15 attributes and ten alternatives are selected and used by experts for the evaluation process of the presented case study. The 15 attributes consist of subjective and objective attributes.

## The ordinal priority approach (OPA)

In 2020, the ordinal priority approach (OPA) was proposed by Ataei et al. [14] to determine the alternative weights based on a set of attributes and as well to deal with both single and group decision-making. We can summarize the steps of OPA with details using this pseudocode as follows:


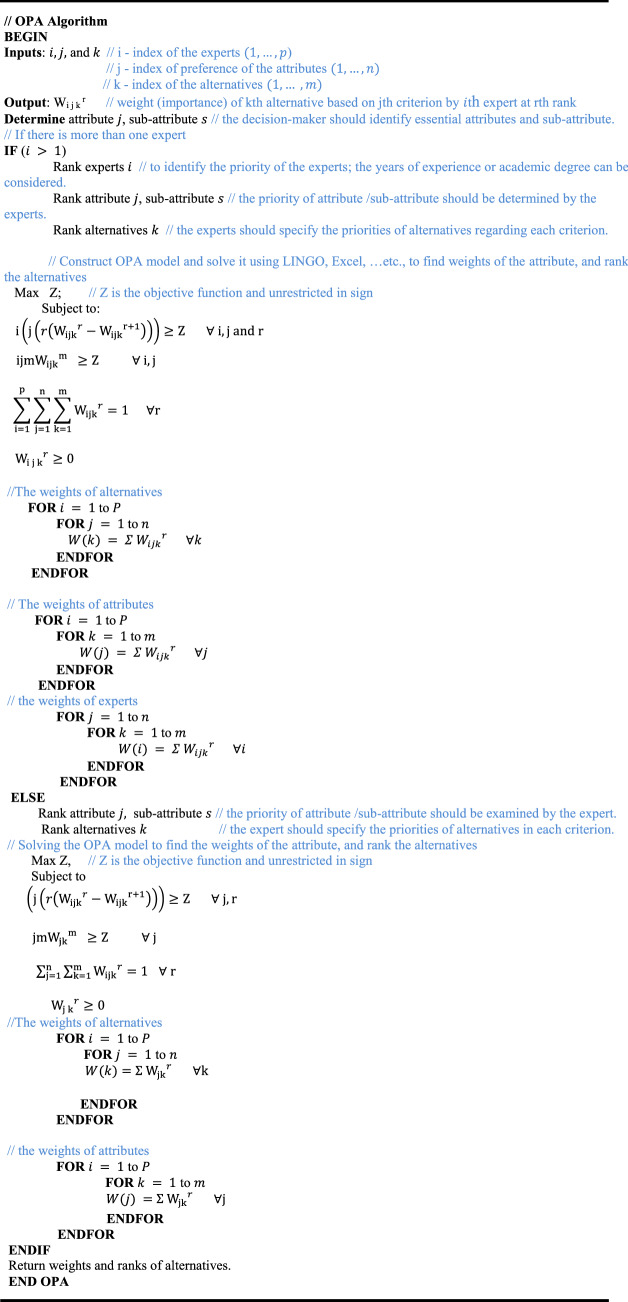


## Neutrosophic ordinal priority approach (OPA-N)

In the current section, the important concepts of the ordinal priority approach in a neutrosophic environment are introduced.

The most significant feature which distinguishes the ordinal priority approach over other existing decision-making techniques is its simplicity. Since every decision is a product of human efforts usually a certain degree of incompleteness and imprecision does exist due to vague and inconsistent information which exist usually in reality. Also, almost all decisions originate from subjective ordinal preferences. As an example, when someone wants to buy a new car, he/she does not construct any comparison matrix or make any normalization of data, he/she should only prioritize all available cars regarding the desired attributes such as price, speed, etc. Since almost all decisions based on linguistic variables as high, very high these values have vague information also. For example, when a father says that he bought a very good bike for his daughter, but his daughter's opinion about the bike is not very good. Therefore, handling uncertainty using linguistic variables is also not enough. Also, decision-makers usually in reality when they give their opinions about a statement may say that this statement is 60% true, 50% false, and 20% not sure.

Therefore, the neutrosophic set is the best concept for handling all the previous problems, since it is able to handle vague and inconsistent information which exists usually in the decision-making process [32], and is also able to deal with paraconsistent information in contrast to classical and fuzzy sets. Since OPA has not yet been presented using neutrosophic, we were motivated to extend it in the neutrosophic environment (OPA-N) for the first time.

The OPA-N goes to transform linguistic information into triangular neutrosophic numbers using a newly presented neutrosophic scale. We should note that this approach can be used for group and individual decision-making problems.

**Fig. 1:**
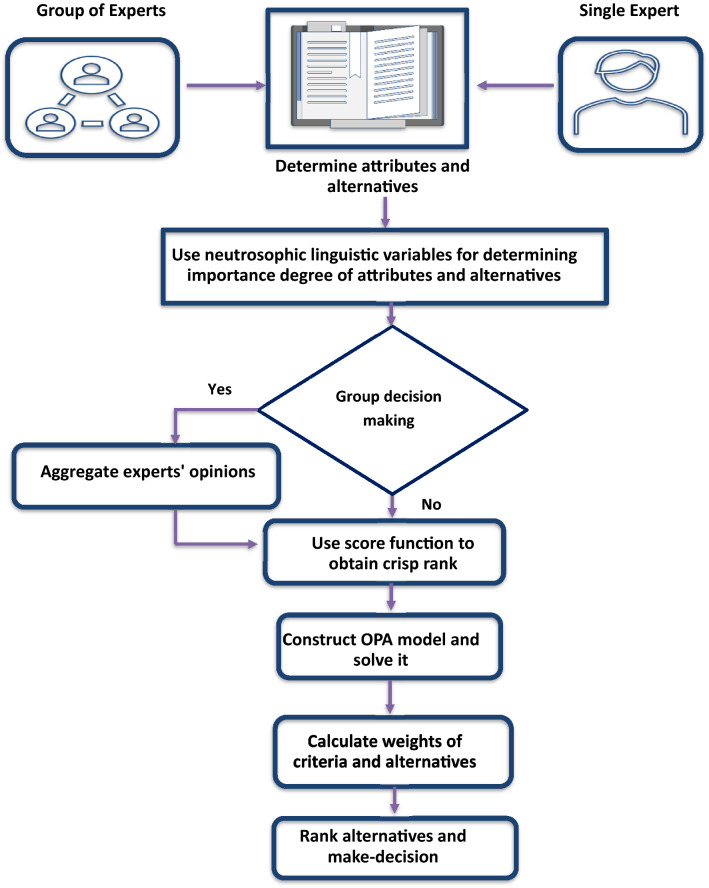
Flowchart of neutrosophic OPA

The flowchart of the OPA-N is presented in Fig. 1. Also, the suggested OPA-N involves some simple steps that have been delineated in the following steps:

*Step 1:* Select experts according to the problem domain. Since experts are persons with both knowledge and experience in a very high-level domain, in our algorithm, we considered all experts to have the same important degree and then does not prioritize them. Also, the process in which classical OPA prioritizes experts based on the organizational chart may be not sufficient in some cases in reality, and if the prioritization process is not precise, then the outcome decision will be affected.

*Step 2:* Let experts begin to determine attributes regarding their opinions and also available alternative alternatives.

*Step 3: *To calculate the relative importance of attributes, use the scale presented in Table 2.

**Table 2:** Linguistic variables for determining the importance degree of attributes and alternatives

Linguistic terms	The lower, median, upper values of triangular numbers (L, M, U)	Confirmation degree of expert opinion (CD)
Absolutely not important	$\langle (0, 0, 0)\rangle$	Absolutely not sure (0,1,1)
Not important	$\langle (0, \mathrm{0,1})\rangle$	Not sure (0.25,0.75, 0.75)
Slightly important	$\langle (1, 2, 3)\rangle$	Slightly sure ($0.45 ,0.60, 0.60$)
Median important	$\langle (2, \mathrm{3,4})\rangle$	Median sure ($0.50 ,0.50, 0.50$)
Important	$\langle (3, \mathrm{4,5})\rangle$	Sure ($0.75 ,0.20, 0.20$)
Strongly important	$\langle (5, 6, 7)\rangle$	Strongly sure$(0.85 ,0.15, 0.15$)
Very strongly important	$\langle (6, 7, 8)\rangle$	Very strongly sure ($0.90 ,0.10, 0.10$)
Absolutely important	$\langle (7, 8, 9)\rangle$	Absolutely sure ($1.00 ,0.00, 0.0$)

*Step 4:* If we have more than one expert make an aggregation of their opinions. The aggregation operator (G) means a mapping function denoted as $G:{\psi }^{n}\to\uppsi$. Here, in this aggregation method, we calculate the average value of the importance degree of each attribute via the dividing sum of the relative importance of the $j$ attribute by the $k$ expert or decision-maker on $n .$ Since $n$ is the number of experts or decision-makers.

From Table 2, the expert or decision-maker must make the rank of attributes and alternatives in the form of triangular neutrosophic numbers ($L, M, U$; CD); as an example, if the expert will rank the first alternative as the best one and give it an “Absolutely important” linguistic variable and the degree of confirmation of her/his opinion about the rank of the first alternative is very strongly sure, then the final evaluation value will take the following form of the triangular neutrosophic number $\langle \left(\mathrm{7,8},9\right);0.90 ,0.10, 0.10\rangle ,$ where the first part $(\mathrm{7,8},9)$ is lower, median, and upper bound for triangular neutrosophic number, ($0.90,0.10, 0.10$) is the confirmation degree which consists of maximum truthiness value, minimum indeterminacy, and falsity degrees of a triangular number.

*Step 5:* After making an aggregation of experts' opinions about the importance of attributes uses the score function equation for obtaining the final rank or priority of aggregated values as follows:

Let ${\tilde{A }}_{1}$=$\langle ({A}_{1}, {A}_{2}, {A}_{3});{\mu }_{{\tilde{A }}_{1}} ,{\gamma }_{{\tilde{A }}_{1}}, {\uplambda }_{{\tilde{A }}_{1}}\rangle$ be a triangular neutrosophic number then the score function equals

1 $$S\left({\tilde{A }}_{1}\right)=\frac{1}{12}({A}_{1}+ {2A}_{2}+ {A}_{3})*\left[2+{\mu }_{{\tilde{A }}_{1}}-{\gamma }_{{\tilde{A }}_{1}}-{\uplambda }_{{\tilde{A }}_{1}}\right].$$

From obtained score function of the attribute, if $S\left({\tilde{C }}_{1}\right)$ $> S\left({\tilde{C }}_{2}\right)$, then ${\tilde{C }}_{1}>{\tilde{C }}_{2}$, which means that ${\tilde{C }}_{1}$ will take the first priority and ${\tilde{C }}_{2}$ will take second priority. Also, if $S({\tilde{C }}_{1})<S({\tilde{C }}_{2})$, then ${\tilde{C }}_{1}<{\tilde{C }}_{2}$, and if $S\left({\tilde{C }}_{1}\right)=S({\tilde{C }}_{2})$, then ${\tilde{C }}_{1}={\tilde{C }}_{2}$.

*Step 6:* After determining the final rank of attributes via considering all aspects of uncertainty and simulating the real decision-making process, use Table 2 also to determine the relative importance of alternatives according to each criterion and also the previous score function for prioritizing them. If your system has more than one expert, repeat step 4 and use Eq. (1) for obtaining the final rank of aggregated value.

*Step 7:* Now, we can traditionally present variables, sets, and indexes of the OPA model as appeared in Table 3 and solve it as follows:

**Table 3:** Ordinal priority approach's sets, indexes, and variables

Sets	
$C$	Set of attributes
$A$	Set of alternatives (robots)
Indexes	
$c$	Index of attributes preferences $(\mathrm{1,2},\dots ,n)$
$a$	Index of alternatives $(\mathrm{1,2},\dots ..,m)$
Variables	
$Z$	Objective function
${A}_{ca}^{r}$	The $a$ th alternative is based on attribute $c$ at rank $r$
${W}_{ca}^{r}$	Weight of alternative $a$ th based on attribute $c$ th at the $rth$ rank

For expressing rank of alternatives based on various values of $c$, the equation will take this form

2 $${A}_{ca}^{1}\ge {A}_{ca}^{2}\ge \dots \ge {A}_{ca}^{r}\ge {A}_{ca}^{r+1}\ge \dots {\ge A}_{ca}^{m}\forall \mathrm{ c},\mathrm{ a}.$$

For the superiority of ${i}^{th}$ alternative over ${l}^{th}$ alternative, we also have the relationship as follows:

3 $${W}_{ca}^{1}\ge {W}_{ca}^{2}\ge \dots \ge {W}_{ca}^{r}\ge {W}_{ca}^{r+1}\ge {\dots \ge W}_{ca}^{n}\forall \mathrm{ c},\mathrm{ a}.$$

From Eq. (3) we can obtain relation among ${W}_{ca}^{r}$ and ${W}_{ca}^{r+1}$ as follows:

$${W}_{ca}^{1}-{W}_{ca}^{2}\ge 0,$$

4 $${W}_{ca}^{2}-{W}_{ca}^{3}\ge 0,$$

$${W}_{ca}^{r}-{W}_{ca}^{r+1}\ge 0,$$

$${W}_{ca}^{m-1}-{W}_{ca}^{m}\ge 0.$$

For presenting the degree of importance of alternatives weights, we will multiply both sides of Eq. (4) by c with rank r as follows:

5 $$c\left(r\left({W}_{ca}^{r}-{W}_{ca}^{r+1}\right)\right)\ge 0.$$

To determine alternative weights, we should solve the following model, in which we want to maximize alternative preference for each attribute:

$$Max\left\{c\left(r\left({W}_{ca}^{r}-{W}_{ca}^{r+1}\right)\right),cm{W}_{ca}^{m}\right\}$$

Subject to:

6 $$\sum_{c=1}^{n}\sum_{a=1}^{m}{W}_{ca}=1$$

$${W}_{ca}\ge $$ 0.

Since we want to maximize the minimization objectives of the model (6), because it is a multi-objective and nonlinear model and then it is as follows:

$$Max Min \left\{c\left(r\left({W}_{ca}^{r}-{W}_{ca}^{r+1}\right)\right),cm{W}_{ca}^{m}\right\}$$

subject to:

7 $$\sum_{c=1}^{n}\sum_{a=1}^{m}{W}_{ca}=1$$

$${W}_{ca}^{r}\ge $$ 0.

For transforming model (7) to its linear form, it will be as follows:

$$Max Z$$

subject to:

8 $$c\left(r\left({W}_{ca}^{r}-{W}_{ca}^{r+1}\right)\right)\ge Z$$

$$cm{W}_{ca}^{m}\ge Z$$

$$\sum_{c=1}^{n}\sum_{a=1}^{m}{W}_{ca}=1$$

$${W}_{ca}\ge $$ 0,

where $Z=$ $Min\left\{c\left(r\left({W}_{ca}^{r}-{W}_{ca}^{r+1}\right)\right),cm{W}_{ca}^{m}\right\}$, and unrestricted in sign.

The obtained model can be solved by different software such as LINGO, Excel, etc.

The weights of alternatives and attributes are determined as follows:

9 $${W}_{a}=\sum_{c=1}^{n}{W}_{ca} \forall \mathrm{ a},$$

10 $${W}_{c}=\sum_{a=1}^{m}{W}_{ca} \forall \mathrm{ c}.$$

*Step 8:* Finally rank alternatives and take a suitable decision.

## Case study: results and analysis

For evaluating the validity of the neutrosophic OPA model, a real case study for selecting the best robot for a new pharmaceutical city “Gypto pharma” in Qalyubia, Egypt is presented and solved.

This city in our case study desires to increase productivity and makes the process of production simpler and empty from human errors by adopting a robotic system. The process of production includes a sequence of cycle elements and tasks. Therefore, this city needs a robot for doing a repetitive job. The robot will do various operations such as producing medicines and vaccines for patients with cancer, coronavirus, brain, nerves, etc. It also will load and unload machines and materials. This city will produce 150 types of drugs and 150 million packages. This number of packages will increase more and more by implementing a suitable robot for this city. This city will produce a new drug that did not produce before in Egypt. Using a suitable robot, this city will become more important in the Middle East and Africa. This new city aims to export pharmaceuticals to cities in Africa. Therefore, selecting the best robots is an essential and difficult task here.

**Fig. 2:**
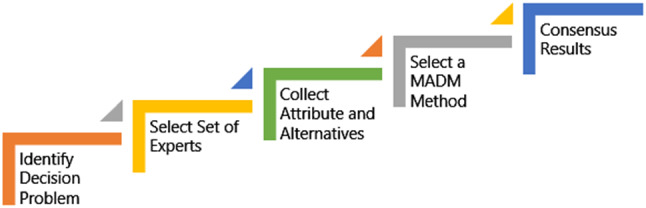
Decision-making methodology

The methodology of the decision-making process consists of the following steps, as shown in Fig 2.

By applying the proposed method in this research for selecting an appropriate robot, the steps are as follows:

*Step 1:* The group of experts is selected by domain problem as in Table 4:

**Table 4:** Information about experts

Expert	Degree	Field
E1	PhD	Artificial intelligence
E2	PhD	Data science
E3	M.Sc	Management consultant
E4	M.Sc	Medicine
E5	PhD	Mechanical engineering

*Step 2:* The attributes are collected from previous research by experts [2, 33–35]. Fifteen attributes and ten robots (alternatives) are selected in this work. Table 5 shows the attributes and their description. As shown in Table 5, there are four objective attributes (C_5_, C_8_, C_11_, and C_14_) and all others are subjective attributes (11 subjective attributes).

**Table 5:** Attributes and its description

Attribute	Description
C_1_: Man–machine interface	More flexible programming and control robots
C_2:_ Work independently	Capable to work independently
C_3_: Programming flexibility	Flexible of function programming
C_4_: Big data	Big data processing capability/memory capacity
C_5_: Velocity	Speed of work
C_6:_ Performance	Environment performance
C_7_: Robot structure	Robot size, weights, materials
C_8:_ Repeatability	Perform some tasks many times
C_9_: Life expectancy	Life expectancy robot
C_10:_ Security and safety	Safety of robot and less risk for preventing control by a hacker
C_11:_ Accuracy	Accuracy for doing the task
C_12_: Communication and behavior interaction	Communication with environment
C_13:_ Sensor	Use sensor fashion for IoT system
C_14:_ Total cost	Purchase, the initial cost
C_15:_ Cloud	Robot manipulating and can use cloud during operation

*Step 3: *Let five experts begin to assess the attributes listed in Table A.1 by using the presented scale in Table 2.

*Step 4:* After assessing attributes by experts, there are five opinions. After that those five opinions were combined to obtain an aggregated opinion by applying the aggregation method.

*Step 5:* After aggregating the five opinions of experts and applying the score function using Eq. (1), the final rank of attributes according to score value is presented in Table 6.

**Table 6:** The neutrosophic computations of attributes for the ranking process

Attribute	Sum of the relative importance of attributes	Score value of average relative importance of attributes	Rank
C_1_	((18,23,28);0.50,0.50,0.50)	2.30	6
C_2_	((19,24,29);0.45,0.60,0.60)	2.00	10
C_3_	((22,27,32);0.45,0.60,0.60)	2.25	7
C_4_	((15,20,25);0.45,0.60,0.60)	1.66	13
C_5_	((21,26,31);0.45,0.60,0.60)	2.17	8
C_6_	((19,24,29);0.50,0.50,0.50)	2.40	5
C_7_	((12,17,22);0.45,0.60,0.60)	1.42	14
C_8_	((24,29,34);0.50,0.50,0.50)	2.90	4
C_9_	((9,14,19);0.45,0.60,0.60)	1.17	15
C_10_	((26,31,36);0.75,0.20,0.20)	4.86	2
C_11_	((23,28,33);0.75,0.20,0.20)	4.39	3
C_12_	((16,21,26);0.45,0.60,0.60)	1.75	12
C_13_	((18,23,28);0.45,0.60,0.60)	1.92	11
C_14_	((29,34,39);0.85,0.15,0.15)	5.78	1
C_15_	((20,25,30);0.45,0.60,0.60)	2.08	9

Experts assess attributes and alternatives in Table A.2.

The combined opinions of experts’ data for alternatives (robots) regarding each attribute are presented in Table A.3. Based on Table A.3, we will take the average of these combined values by dividing it by the number of experts. For determining the final rank of alternatives, we will apply Eq. (1) to the average values of these alternatives. The alternative with the biggest score value will take rank 1, and so on. The final rank of attributes and alternatives are presented in Table 7.

**Table 7:** The final rank of alternatives and attribute

Attribute	Rank attribute	Rank of robots									
		A_1_	A_2_	A_3_	A_4_	A_5_	A_6_	A_7_	A_8_	A_9_	A_10_
C_14_	1	5	8	1	9	3	10	4	2	7	6
C_10_	2	10	2	7	8	9	3	5	1	6	4
C_11_	3	10	9	1	7	2	6	5	3	4	8
C_8_	4	1	10	5	3	6	9	8	7	4	2
C_6_	5	3	5	10	1	7	8	9	4	2	6
C_1_	6	6	4	7	2	10	3	1	9	5	8
C_3_	7	8	6	5	2	7	3	10	4	1	9
C_5_	8	8	5	3	10	1	4	7	6	9	2
C_15_	9	10	1	5	4	2	8	6	3	9	7
C_2_	10	1	2	5	7	4	6	8	10	9	3
C_13_	11	10	2	9	1	8	6	5	3	7	4
C_12_	12	4	2	8	6	1	7	5	3	9	10
C_4_	13	4	8	5	7	2	9	1	10	6	3
C_7_	14	5	8	9	4	2	7	1	10	3	6
C_9_	15	9	6	5	8	4	10	3	1	7	2

The model of OPA_N in Appendix B.

The weights of attributes and alternatives can obtain using the LINGO or MATLAB software. In this work, the results were obtained by the LINGO software.

**Fig. 3:**
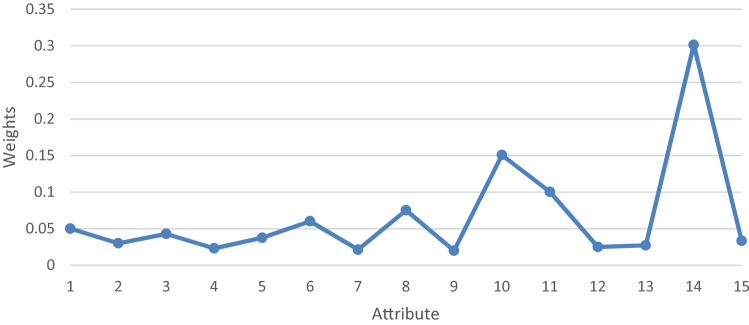
Final weights of attributes

Obtained results show that C14 (i.e., Total Cost) is the attribute with the highest weight and the C9 (i.e., Life Expectancy) is the attribute with the least weight, as shown in Fig. 3.

**Fig. 4:**
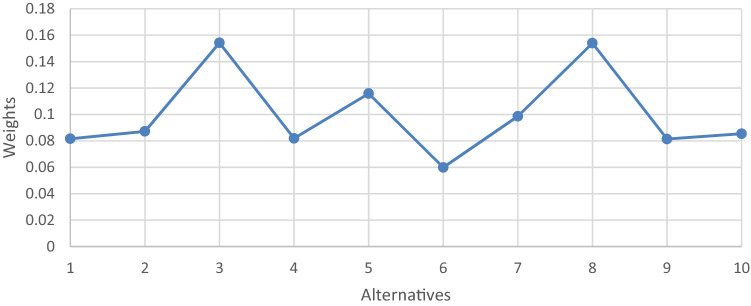
Final Weights of alternatives

According to the final rank of robots (alternatives), A_3_ is the best robot and A_6_ is the worst robot as appears in Fig. 4.

## Sensitivity analysis

The sensitivity analysis of the OPA-N results is conducted to assess the persistence of the priority rating and it can be an efficient way to determine the proposed approach’s efficiency.

The rank of attributes was subjected to a sensitivity analysis. Therefore, we will show how various priorities of attributes will impact on final rank of alternatives.

As we have 15 attributes, there are many cases, so we take only 19 random cases as in Table 8.

**Table 8:** The case of change in priority of attributes

Case #	C_14_	C_10_	C_11_	C_8_	C_6_	C_1_	C_3_	C_5_	C_15_	C_2_	C_13_	C_12_	C_4_	C_7_	C_9_
1	C_11_	C_13_	C_10_	C_2_	C_6_	C_1_	C_3_	C_5_	C_15_	C_14_	C_8_	C_12_	C_4_	C_7_	C_9_
2	C_9_	C_7_	C_4_	C_12_	C_13_	C_2_	C_15_	C_5_	C_3_	C_1_	C_6_	C_8_	C_11_	C_10_	C_14_
3	C_10_	C_6_	C_11_	C_1_	C_8_	C_9_	C_5_	C_3_	C_2_	C_15_	C_12_	C_4_	C_13_	C_14_	C_7_
4	C_1_	C_2_	C_3_	C_4_	C_5_	C_6_	C_7_	C_8_	C_9_	C_10_	C_11_	C_12_	C_13_	C_14_	C_15_
5	C_2_	C_4_	C_6_	C_8_	C_10_	C_12_	C_14_	C_1_	C_3_	C_5_	C_7_	C_9_	C_11_	C_13_	C_15_
6	C_1_	C_3_	C_5_	C_7_	C_9_	C_11_	C_13_	C_15_	C_2_	C_4_	C_6_	C_8_	C_10_	C_12_	C_14_
7	C_3_	C_6_	C_9_	C_12_	C_15_	C_2_	C_5_	C_8_	C_11_	C_14_	C_1_	C_4_	C_7_	C_10_	C_13_
8	C_4_	C_8_	C_12_	C_3_	C_7_	C_11_	C_15_	C_2_	C_6_	C_10_	C_14_	C_1_	C_5_	C_9_	C_13_
9	C_5_	C_10_	C_15_	C_4_	C_9_	C_14_	C_3_	C_8_	C_13_	C_2_	C_7_	C_12_	C_1_	C_6_	C_11_
10	C_6_	C_12_	C_5_	C_11_	C_4_	C_10_	C_3_	C_9_	C_15_	C_2_	C_8_	C_14_	C_1_	C_7_	C_13_
11	C_7_	C_14_	C_6_	C_13_	C_5_	C_12_	C_4_	C_11_	C_3_	C_10_	C_2_	C_9_	C_1_	C_8_	C_15_
12	C_8_	C_7_	C_15_	C_6_	C_14_	C_5_	C_13_	C_4_	C_12_	C_3_	C_11_	C_2_	C_10_	C_1_	C_9_
13	C_9_	C_8_	C_7_	C_6_	C_15_	C_5_	C_14_	C_4_	C_13_	C_3_	C_12_	C_2_	C_11_	C_1_	C_10_
14	C_10_	C_9_	C_8_	C_7_	C_6_	C_5_	C_15_	C_4_	C_14_	C_3_	C_13_	C_2_	C_12_	C_1_	C_11_
15	C_11_	C_10_	C_9_	C_8_	C_7_	C_6_	C_5_	C_4_	C_15_	C_3_	C_14_	C_2_	C_13_	C_1_	C_12_
16	C_12_	C_11_	C_10_	C_9_	C_8_	C_7_	C_6_	C_5_	C_4_	C_3_	C_15_	C_2_	C_14_	C_1_	C_13_
17	C_13_	C_12_	C_11_	C_10_	C_9_	C_8_	C_7_	C_6_	C_5_	C_4_	C_3_	C_2_	C_15_	C_1_	C_14_
18	C_14_	C_13_	C_12_	C_11_	C_10_	C_9_	C_8_	C_7_	C_6_	C_5_	C_4_	C_3_	C_2_	C_1_	C_15_
19	C_15_	C_14_	C_13_	C_12_	C_11_	C_10_	C_9_	C_8_	C_7_	C_6_	C_5_	C_4_	C_3_	C_2_	C_1_

**Fig. 5:**
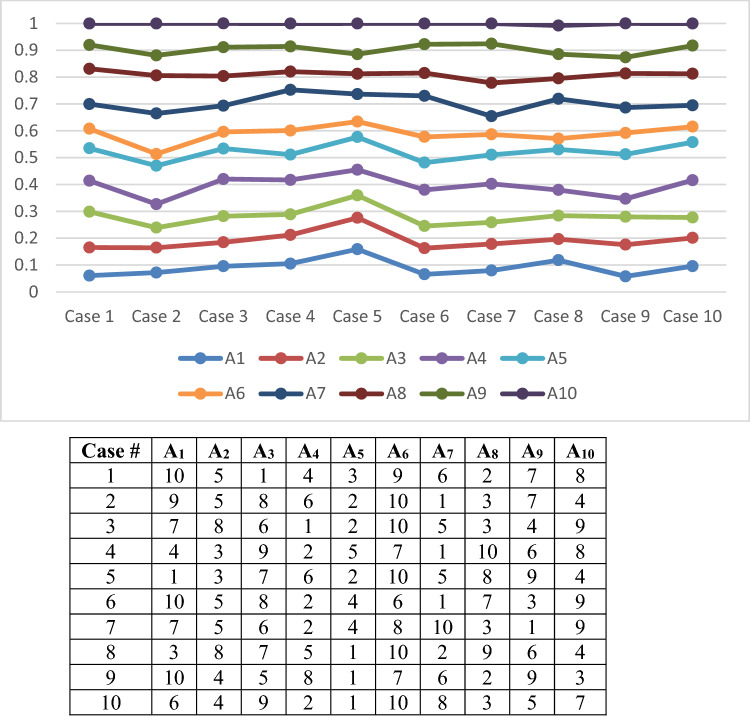
Different weights of alternatives under sensitivity analysis from cases 1–10

**Fig. 6:**
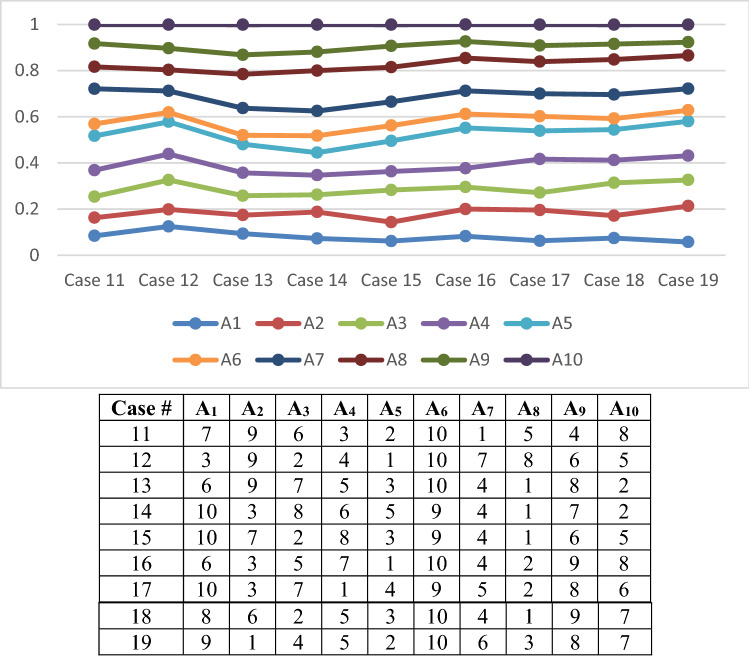
Different weights of alternatives under sensitivity analysis from cases 11–19

Figures 5 and 6 show the weights of alternatives based on various ranks of attributes. As seen in the figures, adjusting the ranking of the attributes has a direct influence on the weights of the alternatives.

The findings of the sensitivity analysis indicate that in cases 8, 10, 12, and 16, A_5_ is the best alternative, and A_6_ is the worst one. In cases 2 and 11, A_7_ is the best robot and A_6_ is the worst, and also, in cases 13 and 18, A_8_ is the best alternative and A_6_ is the worst one. Also, in cases 14, and 15, A_8_ is the best alternative, and A_1_ is the worst one. Therefore, we can conclude that A_5_ appeared five times as the best alternative. Also, A_7_ and A_8_ appeared four times as the best alternative. A_4_ appeared also two times as the best. Finally, A_3_, A_1_, A_9_, A_2_ appeared only one time as the best alternative in the nineteen cases. However, as the lowest rank, A_6_ appeared eleven times, A_1_ appeared six times, A_7_ and A_8_ appeared one time as the lowest rank in 19 cases.

## Comparative analysis

In this section, we mutually compared the obtained results of the proposed algorithm (OPA-N) with obtained results of fuzzy OPA (OPA-F) which was presented in [21].

**Fig. 7:**
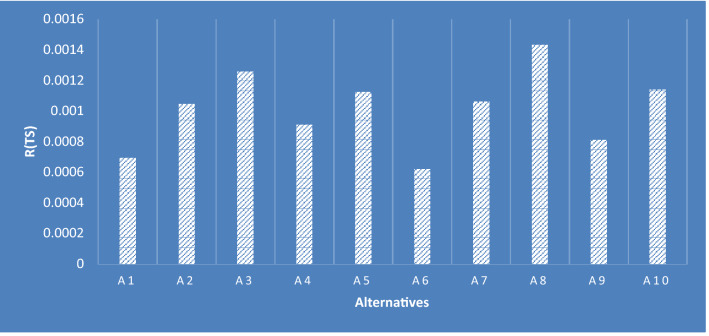
The rank of alternatives based on defuzzification formula R (TS)

By solving our case study using (OPA-F) which was presented in [21], the results showed that alternative 8 is the best one as appears in Table 9 and Fig. 7. The total rank of alternatives is as follows: ${A}_{8}>{A}_{3}>{A}_{10}>{A}_{5}>{A}_{7}>{A}_{2}>{A}_{4}>{A}_{9}>{A}_{1}>{A}_{6}.$

**Table 9:** Total fuzzy score of each alternative

Alternatives	L	M	U	R(TS)	Rank
A1	0.0005419376	0.0006777076	0.0009085798	0.000694	9
A2	0.0008219997	0.0010786460	0.0011490333	0.001048	6
A3	0.0009490061	0.0012981143	0.0014115375	0.001259	2
A4	0.0007755645	0.0009122549	0.0010336042	0.00091	7
A5	0.0009542543	0.0011288226	0.0012877669	0.001126	4
A6	0.0004783107	0.0006179901	0.0007868482	0.000623	10
A7	0.0009271659	0.0010670592	0.0011796840	0.001063	5
A8	0.0011350590	0.0013732713	0.0019678994	0.001433	1
A9	0.0006663868	0.0008009228	0.0010163652	0.000814	8
A10	0.0009077259	0.0011758927	0.0012348042	0.001141	3

For comparing ranks of the proposed approach (OPA-N) with fuzzy OPA (OPA-F), we used the following statistical methods as follows:

- *Spearman's correlation:* it is one of the most important methods for finding whether there is a correlation between two ordinal variables or there isn't. The Spearman's correlation is calculated as follows:
  11 $${S}_{C}=1-\left[\frac{6.\sum_{m=1}^{A}{\left({d}_{m}\right)}^{2}}{A.\left({A}^{2}-1\right)}\right],$$
  where $A$ is the number of alternatives and ${d}_{m}$ is the difference between the two ranks of alternatives. If ${S}_{C}$ has value close to $+1$ or $-1$, it means that it is a strong correlation. However, if the value of ${S}_{C}$ is close to $0$, it means that it is a weak correlation.
- *Person's correlation:* shows the extent to which two variables are linearly correlated. If the correlation coefficient is 1, then there is a total positive linear correlation. However, if the correlation coefficient is -1, then there is a total negative linear correlation. Finally, if the correlation coefficient is 0, then there is no linear correlation. The formula of Person's correlation coefficient is as follows:
  12 $${p}_{cor}\left(b,c\right)=\frac{cov(b,c)}{{\sigma }_{b}{\sigma }_{c}},$$
  where $cov(b,c)$ is the covariance among $b,c$, and ${\sigma }_{b},{\sigma }_{c}$ is the standard deviation of $b$ and $c$, respectively.

The final rank of alternatives using the proposed neutrosophic OPA (OPA-N) approach and fuzzy OPA (OPA-F) approach is presented in Table 10.

**Table 10:** Rank of alternatives based on OPA-N and OPA-F

Alternatives	OPA-N	OPA-F
A1	8	9
A2	5	6
A3	1	2
A4	7	7
A5	3	4
A6	10	10
A7	4	5
A8	2	1
A9	9	8
A10	6	3

Firstly, we calculated Spearman's correlation coefficient for comparing obtained ranks from two approaches. Spearman's correlation coefficient is equal to $0.90303$ which shows the strong correlation between the two approaches.

Also, we calculated the Person's correlation coefficient for showing linear relation among two approaches. The Pearson's correlation coefficient is also equal to $0.90303$ which shows a strong linear correlation among the two approaches. By considering $\alpha =0.05$, then.

$$\mathrm{t}=0.90303\frac{\sqrt{10-2}}{\sqrt{1-{0.90303}^{2}}}=5.94.$$

Since $t=5.94>{t}_{0.05}(10-2)=1.8$, then the hypothesis test shows that there is a strong positive correlation among alternative weights of the proposed method and OPA-F.

Finally, the finding from the comparative study of OPA-F and OPA-N for measuring and ranking the weights of attributes/alternatives are as follows:

- Even though both methods produced near results, the suggested model (OPA-N) is simpler and more useful than the OPA-F for the following reasons:
- To calculate the weights of attributes/alternatives, a crisp model must be generated from the fuzzy model. However, the conversion process from fuzzy model to crisp increases the number of equations by three times. The increase in the number of equations makes the OPA-F model more complex, time, and storage-consuming compared with (OPA-N). Table 11 shows the number of constraints, variables, and iterations for OPA_N and OPA_F.
- To solve this case study using the OPA-F model, we constructed a total of 7452 crisp equations which were needed to obtain the ranks for the alternatives only, so in large-scale problems, the OPA-F model will be more complex, and time-consuming than the proposed OPA-N model.
- Finally, the OPA-F model cannot deal efficiently with vague and inconsistent information which exist usually in the decision-making process, since it considers only the truth-membership degree, while the proposed model is capable of handling vague and inconsistent information efficiently and simulating natural human thinking during the decision-making process via considering truth, indeterminacy, and falsity membership degrees.

**Table 11:** Difference between OPA_N and OPA-F at solving the same case study

Case study components	OPA_N	OPA_F [21]
Number of experts	5	5
Number of attributes	15	15
Number of alternatives	10	10
Number of total variables	178	4966
Number of total constraints	329	7452
Number of iterations	0	2107

The difference between OPA-N and OPA-F at solving the same case study is presented in Table 11.

## Managerial implications

Since companies need robots for doing complex and repetitive tasks, they need to select appropriate types for fulfilling their missions in the best manner. As the selection process is a hard task due to several conflicting attributes which exist nowadays, then we need an ordering technique that commonly contains various extents of selection. In this research, we presented for the first time the ordinal priority approach in the neutrosophic environment for handling uncertainty which exist usually in the selection process. The proposed OPA_N proved its applicability to deal with subjective and objective attributes under uncertainty for evolving a strong decision.

The suggested model can be a powerful guide for companies or organization that desire to use a robotic system in hospitals, pharmacy, manufacturing company, and companies that wishes to robotize the welding section on their manufacturing units. In addition, governments can use the suggested model for making precise decisions about any social, economic, and environmental problems.

## Conclusions and future directions

In this research, a new extension of the OPA method is presented in the neutrosophic environment for robot selection problems. This research presented for the first time a case study of a new pharmaceutical city in Egypt for selecting the best robot from among available alternatives for increasing productivity of pharmaceutical and to serve Egyptian people. In this case, we selected five experts from various specialties. The experts constructed the evaluation process of robots based on 15 attributes and 10 alternatives.

The finding of the outcomes illustrated that the proposed method is able to handle uncertainty efficiently. Also, the proposed method is simpler and more helpful than the classical and fuzzy model of OPA. Not only this, but by comparing OPA_N with OPA-F, we concluded that OPA-N is less time-consuming than OPA-F as fuzzy OPA produces a huge number of equations on a large scale. On the other hand, some limitations must be enhanced in the further study such as considering interrelationships among attributes and not only independent attributes. Also, we need to develop a proposed method to consider in detail the impact of positive and negative attributes on the final decision.

In the future, we plan to use various multi-attribute decision-making methods and present them in a neutrosophic environment using the alpha cut method to solve the problem of selecting the robot with more difficult and complex dependencies between attributes.

## Supplementary Information

Below is the link to the electronic supplementary material.

Supplementary file1 (DOCX 69 kb)
