# Supplementary material for: New extension of ordinal priority approach for multiple attribute decision-making problems: design and analysis
Source: Complex Intell Systems. 2022 Apr 29;8(6):4955–70. doi: 10.1007/s40747-022-00721-w (PMC9051802; doi:10.1007/s40747-022-00721-w)
Supplement: Supplementary file 1 — Supplementary file1 (DOCX 69 kb) [file 40747_2022_721_MOESM1_ESM.docx]

**Appendix A**

**Table A.1:** Evaluation values of attributes according to each expert

| **Attribute/Experts** | **1** | **2** | **3** | **4** | **5** | **Attribute/Expert** | **1** | **2** | **3** | **4** | **5** |
| --- | --- | --- | --- | --- | --- | --- | --- | --- | --- | --- | --- |
| C_1_ | ((3,4,5);0.75,0.20,0.20) | ((2,3,4);0.50,0.50,0.50) | ((6,7,8);0.9,0.10,0.10) | ((5,6,7);0.85,0.15,0.15) | ((2,3,4);0.50,0.50,0.50) | C_6_ | ((2,3,4);0.50,0.50,0.50) | ((3,4,5);0.75,0.20,0.20) | ((5,6,7);0.85,0.15,0.15) | ((3,4,5);0.75,0.20,0.20) | ((6,7,8);0.9,0.10,0.10) |
| C_2_ | ((7,8,9);1.00,0.0,0.0) | ((6,7,8);0.9,0.10,0.10) | ((1,2,3);0.45,0.60,0.60) | ((2,3,4);0.50,0.50,0.50) | ((3,4,5);0.75,0.20,0.20) | C_7_ | ((5,6,7);0.85,0.15,0.15) | ((1,2,3);0.45,0.60,0.60) | ((2,3,4);0.50,0.50,0.50) | ((1,2,3);0.45,0.60,0.60) | ((3,4,5);0.75,0.20,0.20) |
| C_3_ | ((1,2,3);0.45,0.60,0.60) | ((5,6,7);0.85,0.15,0.15) | ((3,4,5);0.75,0.20,0.20) | ((7,8,9);1.00,0.0,0.0) | ((6,7,8);0.9,0.10,0.10) | C_8_ | ((6,7,8);0.9,0.10,0.10) | ((6,7,8);0.9,0.10,0.10) | ((7,8,9);1.00,0.0,0.0) | ((3,4,5);0.75,0.20,0.20) | ((2,3,4);0.50,0.50,0.50) |
| C_4_ | ((6,7,8);0.9,0.10,0.10) | ((1,2,3);0.45,0.60,0.60) | ((2,3,4);0.50,0.50,0.50) | ((1,2,3);0.45,0.60,0.60) | ((5,6,7);0.85,0.15,0.15) | C_9_ | ((1,2,3);0.45,0.60,0.60) | ((3,4,5);0.75,0.20,0.20) | ((1,2,3);0.45,0.60,0.60) | ((3,4,5);0.75,0.20,0.20) | ((1,2,3);0.45,0.60,0.60) |
| C_5_ | ((3,4,5);0.75,0.20,0.20) | ((7,8,9);1.00,0.0,0.0) | ((3,4,5);0.75,0.20,0.20) | ((7,8,9);1.00,0.0,0.0) | ((1,2,3);0.45,0.60,0.60) | C_10_ | ((5,6,7);0.85,0.15,0.15) | ((6,7,8);0.9,0.10,0.10) | ((7,8,9);1.00,0.0,0.0) | ((3,4,5);0.75,0.20,0.20) | ((5,6,7);0.85,0.15,0.15) |
| **Attribute/Experts** | **1** | **2** | **3** | **4** | **5** | **Attribute/Expert** | **1** | **2** | **3** | **4** | **5** |
| C_11_ | ((3,4,5);0.75,0.20,0.20) | ((5,6,7);0.85,0.15,0.15) | ((5,6,7);0.85,0.15,0.15) | ((5,6,7);0.85,0.15,0.15) | ((5,6,7);0.85,0.15,0.15) | C_14_ | ((6,7,8);0.9,0.10,0.10) | ((7,8,9);1.00,0.0,0.0) | ((6,7,8);0.9,0.10,0.10) | ((5,6,7);0.85,0.15,0.15) | ((5,6,7);0.85,0.15,0.15) |
| C_12_ | ((2,3,4);0.50,0.50,0.50) | ((5,6,7);0.85,0.15,0.15) | ((3,4,5);0.75,0.20,0.20) | ((1,2,3);0.45,0.60,0.60) | ((5,6,7);0.85,0.15,0.15) | C_15_ | ((3,4,5);0.75,0.20,0.20) | ((5,6,7);0.85,0.15,0.15) | ((5,6,7);0.85,0.15,0.15) | ((6,7,8);0.9,0.10,0.10) | ((1,2,3);0.45,0.60,0.60) |
| C_13_ | ((3,4,5);0.75,0.20,0.20) | ((1,2,3);0.45,0.60,0.60) | ((5,6,7);0.85,0.15,0.15) | ((3,4,5);0.75,0.20,0.20) | ((6,7,8);0.9,0.10,0.10) | -------- | | | | | |

**Table A.2:** Evaluation values of alternatives based on each attribute according to each expert

| **Experts** | **Attributes in priority order** | **Robots (alternatives)** | | | | | | | | | |
| --- | --- | --- | --- | --- | --- | --- | --- | --- | --- | --- | --- |
|  |  | **A_1_** | **A_2_** | **A_3_** | **A_4_** | **A_5_** | **A_6_** | **A_7_** | **A_8_** | **A_9_** | **A_10_** |
| 1 | C_14_ | ((5,6,7);0.85,0.15,0.15) | ((3,4,5);0.75,0.20,0.20) | ((7,8,9);1.00,0.0,0.0) | ((7,8,9);1.00,0.0,0.0) | ((3,4,5);0.75,0.20,0.20) | ((7,8,9);1.00,0.0,0.0) | ((5,6,7);0.85,0.15,0.15) | ((5,6,7);0.85,0.15,0.15) | ((7,8,9);1.00,0.0,0.0) | ((3,4,5);0.75,0.20,0.20) |
|  | C_10_ | ((1,2,3);0.40,0.60,0.60) | ((5,6,7);0.85,0.15,0.15) | ((3,4,5);0.75,0.20,0.20) | ((5,6,7);0.85,0.15,0.15) | ((7,8,9);1.00,0.0,0.0) | ((5,6,7);0.85,0.15,0.15) | ((7,8,9);1.00,0.0,0.0) | ((5,6,7);0.85,0.15,0.15) | ((3,4,5);0.75,0.20,0.20) | ((7,8,9);1.00,0.0,0.0) |
|  | C_11_ | ((1,2,3);0.45,0.60,0.60) | ((1,2,3);0.40,0.60,0.60) | ((7,8,9);1.00,0.0,0.0) | ((5,6,7);0.85,0.15,0.15) | ((3,4,5);0.75,0.20,0.20) | ((7,8,9);1.00,0.0,0.0) | ((3,4,5);0.75,0.20,0.20) | ((5,6,7);0.85,0.15,0.15) | ((7,8,9);1.00,0.0,0.0) | ((1,2,3);0.40,0.60,0.60) |
|  | C_8_ | ((6,7,8);0.9,0.10,0.10) | ((1,2,3);0.40,0.60,0.60) | ((5,6,7);0.85,0.15,0.15) | ((5,6,7);0.85,0.15,0.15) | ((5,6,7);0.85,0.15,0.15) | ((1,2,3);0.40,0.60,0.60) | ((1,2,3);0.40,0.60,0.60) | ((3,4,5);0.75,0.20,0.20) | ((5,6,7);0.85,0.15,0.15) | ((7,8,9);1.00,0.0,0.0) |
|  | C_6_ | ((5,6,7);0.85,0.15,0.15) | ((3,4,5);0.75,0.20,0.20) | ((1,2,3);0.40,0.60,0.60) | ((7,8,9);1.00,0.0,0.0) | ((3,4,5);0.75,0.20,0.20) | ((1,2,3);0.40,0.60,0.60) | ((1,2,3);0.40,0.60,0.60) | ((5,6,7);0.85,0.15,0.15) | ((7,8,9);1.00,0.0,0.0) | ((3,4,5);0.75,0.20,0.20) |
|  | C_1_ | ((6,7,8);0.9,0.10,0.10) | ((5,6,7);0.85,0.15,0.15) | ((1,2,3);0.45,0.60,0.60) | ((3,4,5);0.75,0.20,0.20) | ((1,2,3);0.45,0.60,0.60) | ((3,4,5);0.75,0.20,0.20) | ((5,6,7);0.85,0.15,0.15) | ((1,2,3);0.45,0.60,0.60) | ((3,4,5);0.75,0.20,0.20) | ((1,2,3);0.40,0.60,0.60) |
|  | C_3_ | ((5,6,7);0.85,0.15,0.15) | ((3,4,5);0.75,0.20,0.20) | ((3,4,5);0.75,0.20,0.20) | ((6,7,8);0.9,0.10,0.10) | ((1,2,3);0.40,0.60,0.60) | ((3,4,5);0.75,0.20,0.20) | ((3,4,5);0.75,0.20,0.20) | ((3,4,5);0.75,0.20,0.20) | ((5,6,7);0.85,0.15,0.15) | ((3,4,5);0.75,0.20,0.20) |
|  | C_5_ | ((3,4,5);0.75,0.20,0.20) | ((3,4,5);0.75,0.20,0.20) | ((3,4,5);0.75,0.20,0.20) | ((5,6,7);0.85,0.15,0.15) | ((3,4,5);0.75,0.20,0.20) | ((6,7,8);0.9,0.10,0.10) | ((1,2,3);0.40,0.60,0.60) | ((3,4,5);0.75,0.20,0.20) | ((7,8,9);1.00,0.0,0.0) | ((7,8,9);1.00,0.0,0.0) |
|  | C_15_ | ((5,6,7);0.85,0.15,0.15) | ((7,8,9);1.00,0.0,0.0) | ((7,8,9);1.00,0.0,0.0) | ((7,8,9);1.00,0.0,0.0) | ((5,6,7);0.85,0.15,0.15) | ((7,8,9);1.00,0.0,0.0) | ((5,6,7);0.85,0.15,0.15) | ((7,8,9);1.00,0.0,0.0) | ((1,2,3);0.40,0.60,0.60) | ((6,7,8);0.9,0.10,0.10) |
|  | C_2_ | ((6,7,8);0.9,0.10,0.10) | ((7,8,9);1.00,0.0,0.0) | ((3,4,5);0.75,0.20,0.20) | ((5,6,7);0.85,0.15,0.15) | ((3,4,5);0.75,0.20,0.20) | ((7,8,9);1.00,0.0,0.0) | ((1,2,3);0.40,0.60,0.60) | ((1,2,3);0.40,0.60,0.60) | ((1,2,3);0.40,0.60,0.60) | ((3,4,5);0.75,0.20,0.20) |
|  | C_13_ | ((3,4,5);0.75,0.20,0.20) | ((7,8,9);1.00,0.0,0.0) | ((5,6,7);0.85,0.15,0.15) | ((6,7,8);0.9,0.10,0.10) | ((7,8,9);1.00,0.0,0.0) | ((3,4,5);0.75,0.20,0.20) | ((5,6,7);0.85,0.15,0.15) | ((5,6,7);0.85,0.15,0.15) | ((1,2,3);0.40,0.60,0.60) | ((7,8,9);1.00,0.0,0.0) |
|  | C_12_ | ((7,8,9);1.00,0.0,0.0) | ((5,6,7);0.85,0.15,0.15) | ((6,7,8);0.9,0.10,0.10) | ((5,6,7);0.85,0.15,0.15) | ((7,8,9);1.00,0.0,0.0) | ((6,7,8);0.9,0.10,0.10) | ((7,8,9);1.00,0.0,0.0) | ((5,6,7);0.85,0.15,0.15) | ((1,2,3);0.40,0.60,0.60) | ((3,4,5);0.75,0.20,0.20) |
|  | C_4_ | ((5,6,7);0.85,0.15,0.15) | ((3,4,5);0.75,0.20,0.20) | ((5,6,7);0.85,0.15,0.15) | ((6,7,8);0.9,0.10,0.10) | ((6,7,8);0.9,0.10,0.10) | ((1,2,3);0.40,0.60,0.60) | ((6,7,8);0.9,0.10,0.10) | ((1,2,3);0.40,0.60,0.60) | ((6,7,8);0.9,0.10,0.10) | ((7,8,9);1.00,0.0,0.0) |
|  | C_7_ | ((3,4,5);0.75,0.20,0.20) | ((7,8,9);1.00,0.0,0.0) | ((3,4,5);0.75,0.20,0.20) | ((7,8,9);1.00,0.0,0.0) | ((6,7,8);0.9,0.10,0.10) | ((7,8,9);1.00,0.0,0.0) | ((6,7,8);0.9,0.10,0.10) | ((1,2,3);0.40,0.60,0.60) | ((5,6,7);0.85,0.15,0.15) | ((7,8,9);1.00,0.0,0.0) |
|  | C_9_ | ((7,8,9);1.00,0.0,0.0) | ((3,4,5);0.75,0.20,0.20) | ((7,8,9);1.00,0.0,0.0) | ((5,6,7);0.85,0.15,0.15) | ((3,4,5);0.75,0.20,0.20) | ((5,6,7);0.85,0.15,0.15) | ((7,8,9);1.00,0.0,0.0) | ((7,8,9);1.00,0.0,0.0) | ((6,7,8);0.9,0.10,0.10) | ((7,8,9);1.00,0.0,0.0) |
| 2 | C_14_ | ((3,4,5);0.75,0.20,0.20) | ((6,7,8);0.9,0.10,0.10) | ((5,6,7);0.85,0.15,0.15) | ((5,6,7);0.85,0.15,0.15) | ((7,8,9);1.00,0.0,0.0) | ((3,4,5);0.75,0.20,0.20) | ((6,7,8);0.9,0.10,0.10) | ((7,8,9);1.00,0.0,0.0) | ((3,4,5);0.75,0.20,0.20) | ((7,8,9);1.00,0.0,0.0) |
|  | C_10_ | ((3,4,5);0.75,0.20,0.20) | ((7,8,9);1.00,0.0,0.0) | ((6,7,8);0.9,0.10,0.10) | ((1,2,3);0.45,0.60,0.60) | ((7,8,9);1.00,0.0,0.0) | ((5,6,7);0.85,0.15,0.15) | ((5,6,7);0.85,0.15,0.15) | ((7,8,9);1.00,0.0,0.0) | ((6,7,8);0.9,0.10,0.10) | ((7,8,9);1.00,0.0,0.0) |
|  | C_11_ | (((3,4,5);0.75,0.20,0.20) | ((7,8,9);1.00,0.0,0.0) | ((5,6,7);0.85,0.15,0.15) | ((7,8,9);1.00,0.0,0.0) | ((5,6,7);0.85,0.15,0.15) | ((3,4,5);0.75,0.20,0.20) | ((7,8,9);1.00,0.0,0.0) | ((5,6,7);0.85,0.15,0.15) | ((7,8,9);1.00,0.0,0.0) | ((5,6,7);0.85,0.15,0.15) |
|  | C_8_ | ((7,8,9);1.00,0.0,0.0) | ((5,6,7);0.85,0.15,0.15) | ((3,4,5);0.75,0.20,0.20) | ((5,6,7);0.85,0.15,0.15) | ((7,8,9);1.00,0.0,0.0) | ((3,4,5);0.75,0.20,0.20) | ((5,6,7);0.85,0.15,0.15) | ((7,8,9);1.00,0.0,0.0) | ((5,6,7);0.85,0.15,0.15) | ((7,8,9);1.00,0.0,0.0) |
|  | C_6_ | ((7,8,9);1.00,0.0,0.0) | ((5,6,7);0.85,0.15,0.15) | ((3,4,5);0.75,0.20,0.20) | ((5,6,7);0.85,0.15,0.15) | ((3,4,5);0.75,0.20,0.20) | ((5,6,7);0.85,0.15,0.15) | ((3,4,5);0.75,0.20,0.20) | ((7,8,9);1.00,0.0,0.0) | ((3,4,5);0.75,0.20,0.20) | ((5,6,7);0.85,0.15,0.15) |
|  | C_1_ | ((5,6,7);0.85,0.15,0.15) | ((3,4,5);0.75,0.20,0.20) | ((6,7,8);0.9,0.10,0.10) | ((3,4,5);0.75,0.20,0.20) | ((5,6,7);0.85,0.15,0.15) | ((3,4,5);0.75,0.20,0.20) | ((6,7,8);0.9,0.10,0.10) | ((3,4,5);0.75,0.20,0.20) | ((5,6,7);0.85,0.15,0.15) | ((6,7,8);0.9,0.10,0.10) |
|  | C_3_ | ((3,4,5);0.75,0.20,0.20) | ((5,6,7);0.85,0.15,0.15) | ((3,4,5);0.75,0.20,0.20) | ((6,7,8);0.9,0.10,0.10) | ((7,8,9);1.00,0.0,0.0) | ((3,4,5);0.75,0.20,0.20) | ((5,6,7);0.85,0.15,0.15) | ((6,7,8);0.9,0.10,0.10) | ((7,8,9);1.00,0.0,0.0) | ((5,6,7);0.85,0.15,0.15) |
|  | C_5_ | ((5,6,7);0.85,0.15,0.15) | ((6,7,8);0.9,0.10,0.10) | ((7,8,9);1.00,0.0,0.0) | ((6,7,8);0.9,0.10,0.10) | ((7,8,9);1.00,0.0,0.0) | ((6,7,8);0.9,0.10,0.10) | ((6,7,8);0.9,0.10,0.10) | ((3,4,5);0.75,0.20,0.20) | ((5,6,7);0.85,0.15,0.15) | ((6,7,8);0.9,0.10,0.10) |
|  | C_15_ | ((1,2,3);0.45,0.60,0.60) | ((7,8,9);1.00,0.0,0.0) | ((5,6,7);0.85,0.15,0.15) | ((3,4,5);0.75,0.20,0.20) | ((5,6,7);0.85,0.15,0.15) | ((1,2,3);0.45,0.60,0.60) | ((5,6,7);0.85,0.15,0.15) | ((3,4,5);0.75,0.20,0.20) | ((5,6,7);0.85,0.15,0.15) | ((1,2,3);0.45,0.60,0.60) |
|  | C_2_ | ((7,8,9);1.00,0.0,0.0) | ((3,4,5);0.75,0.20,0.20) | ((7,8,9);1.00,0.0,0.0) | ((5,6,7);0.85,0.15,0.15) | ((3,4,5);0.75,0.20,0.20) | ((7,8,9);1.00,0.0,0.0) | ((5,6,7);0.85,0.15,0.15) | ((3,4,5);0.75,0.20,0.20) | ((6,7,8);0.9,0.10,0.10) | ((5,6,7);0.85,0.15,0.15) |
|  | C_13_ | ((1,2,3);0.45,0.60,0.60) | ((5,6,7);0.85,0.15,0.15) | ((1,2,3);0.45,0.60,0.60) | ((6,7,8);0.9,0.10,0.10) | ((1,2,3);0.45,0.60,0.60) | ((5,6,7);0.85,0.15,0.15) | ((6,7,8);0.9,0.10,0.10) | ((5,6,7);0.85,0.15,0.15) | ((7,8,9);1.00,0.0,0.0) | ((7,8,9);1.00,0.0,0.0) |
|  | C_12_ | ((3,4,5);0.75,0.20,0.20) | ((7,8,9);1.00,0.0,0.0) | ((6,7,8);0.9,0.10,0.10) | ((3,4,5);0.75,0.20,0.20) | ((7,8,9);1.00,0.0,0.0) | ((7,8,9);1.00,0.0,0.0) | ((3,4,5);0.75,0.20,0.20) | ((6,7,8);0.9,0.10,0.10) | ((3,4,5);0.75,0.20,0.20) | ((5,6,7);0.85,0.15,0.15) |
|  | C_4_ | ((5,6,7);0.85,0.15,0.15) | ((7,8,9);1.00,0.0,0.0) | ((3,4,5);0.75,0.20,0.20) | ((6,7,8);0.9,0.10,0.10) | ((5,6,7);0.85,0.15,0.15) | ((3,4,5);0.75,0.20,0.20) | ((7,8,9);1.00,0.0,0.0) | ((1,2,3);0.40,0.60,0.60) | ((6,7,8);0.9,0.10,0.10) | ((3,4,5);0.75,0.20,0.20) |
|  | C_7_ | ((5,6,7);0.85,0.15,0.15) | ((3,4,5);0.75,0.20,0.20) | ((6,7,8);0.9,0.10,0.10) | ((3,4,5);0.75,0.20,0.20) | ((5,6,7);0.85,0.15,0.15) | ((6,7,8);0.9,0.10,0.10) | ((5,6,7);0.85,0.15,0.15) | ((3,4,5);0.75,0.20,0.20) | ((5,6,7);0.85,0.15,0.15) | ((7,8,9);1.00,0.0,0.0) |
|  | C_9_ | ((3,4,5);0.75,0.20,0.20) | ((6,7,8);0.9,0.10,0.10) | ((3,4,5);0.75,0.20,0.20) | ((6,7,8);0.9,0.10,0.10) | ((7,8,9);1.00,0.0,0.0) | ((3,4,5);0.75,0.20,0.20) | ((6,7,8);0.9,0.10,0.10) | ((7,8,9);1.00,0.0,0.0) | ((7,8,9);1.00,0.0,0.0) | ((6,7,8);0.9,0.10,0.10) |
| 3 | C_14_ | ((7,8,9);1.00,0.0,0.0) | ((6,7,8);0.9,0.10,0.10) | ((7,8,9);1.00,0.0,0.0) | ((1,2,3);0.45,0.60,0.60) | ((6,7,8);0.9,0.10,0.10) | ((3,4,5);0.75,0.20,0.20) | ((7,8,9);1.00,0.0,0.0) | ((6,7,8);0.9,0.10,0.10) | ((3,4,5);0.75,0.20,0.20) | ((7,8,9);1.00,0.0,0.0) |
|  | C_10_ | ((6,7,8);0.9,0.10,0.10) | ((7,8,9);1.00,0.0,0.0) | ((3,4,5);0.75,0.20,0.20) | ((7,8,9);1.00,0.0,0.0) | ((1,2,3);0.45,0.60,0.60) | ((6,7,8);0.9,0.10,0.10) | ((3,4,5);0.75,0.20,0.20) | ((7,8,9);1.00,0.0,0.0) | ((6,7,8);0.9,0.10,0.10) | ((3,4,5);0.75,0.20,0.20) |
|  | C_11_ | ((7,8,9);1.00,0.0,0.0) | ((6,7,8);0.9,0.10,0.10) | ((7,8,9);1.00,0.0,0.0) | ((3,4,5);0.75,0.20,0.20) | ((7,8,9);1.00,0.0,0.0) | ((6,7,8);0.9,0.10,0.10) | ((6,7,8);0.9,0.10,0.10) | ((3,4,5);0.75,0.20,0.20) | ((3,4,5);0.75,0.20,0.20) | ((7,8,9);1.00,0.0,0.0) |
|  | C_8_ | ((6,7,8);0.9,0.10,0.10) | ((3,4,5);0.75,0.20,0.20) | ((6,7,8);0.9,0.10,0.10) | ((6,7,8);0.9,0.10,0.10) | ((3,4,5);0.75,0.20,0.20) | ((6,7,8);0.9,0.10,0.10) | ((6,7,8);0.9,0.10,0.10) | ((3,4,5);0.75,0.20,0.20) | ((6,7,8);0.9,0.10,0.10) | ((6,7,8);0.9,0.10,0.10) |
|  | C_6_ | ((3,4,5);0.75,0.20,0.20) | ((7,8,9);1.00,0.0,0.0) | ((6,7,8);0.9,0.10,0.10) | ((6,7,8);0.9,0.10,0.10) | ((3,4,5);0.75,0.20,0.20) | ((6,7,8);0.9,0.10,0.10) | ((6,7,8);0.9,0.10,0.10) | ((3,4,5);0.75,0.20,0.20) | ((6,7,8);0.9,0.10,0.10) | ((3,4,5);0.75,0.20,0.20) |
|  | C_1_ | ((1,2,3);0.45,0.60,0.60) | ((7,8,9);1.00,0.0,0.0) | ((6,7,8);0.9,0.10,0.10) | ((7,8,9);1.00,0.0,0.0) | ((1,2,3);0.45,0.60,0.60) | ((6,7,8);0.9,0.10,0.10) | ((7,8,9);1.00,0.0,0.0) | ((6,7,8);0.9,0.10,0.10) | ((3,4,5);0.75,0.20,0.20) | ((1,2,3);0.45,0.60,0.60) |
|  | C_3_ | ((1,2,3);0.45,0.60,0.60) | ((5,6,7);0.85,0.15,0.15) | ((7,8,9);1.00,0.0,0.0) | ((5,6,7);0.85,0.15,0.15) | ((3,4,5);0.75,0.20,0.20) | ((6,7,8);0.9,0.10,0.10) | ((1,2,3);0.45,0.60,0.60) | ((6,7,8);0.9,0.10,0.10) | ((5,6,7);0.85,0.15,0.15) | ((1,2,3);0.45,0.60,0.60) |
|  | C_5_ | ((7,8,9);1.00,0.0,0.0) | ((3,4,5);0.75,0.20,0.20) | ((6,7,8);0.9,0.10,0.10) | ((3,4,5);0.75,0.20,0.20) | ((7,8,9);1.00,0.0,0.0) | ((3,4,5);0.75,0.20,0.20) | ((6,7,8);0.9,0.10,0.10) | ((7,8,9);1.00,0.0,0.0) | ((3,4,5);0.75,0.20,0.20) | ((3,4,5);0.75,0.20,0.20) |
|  | C_15_ | ((7,8,9);1.00,0.0,0.0) | ((6,7,8);0.9,0.10,0.10) | ((3,4,5);0.75,0.20,0.20) | ((7,8,9);1.00,0.0,0.0) | ((6,7,8);0.9,0.10,0.10) | ((5,6,7);0.85,0.15,0.15) | ((7,8,9);1.00,0.0,0.0) | ((3,4,5);0.75,0.20,0.20) | ((6,7,8);0.9,0.10,0.10) | ((7,8,9);1.00,0.0,0.0) |
|  | C_2_ | ((7,8,9);1.00,0.0,0.0) | ((6,7,8);0.9,0.10,0.10) | ((7,8,9);1.00,0.0,0.0) | ((1,2,3);0.45,0.60,0.60) | ((5,6,7);0.85,0.15,0.15) | ((1,2,3);0.45,0.60,0.60) | ((6,7,8);0.9,0.10,0.10) | ((7,8,9);1.00,0.0,0.0) | ((1,2,3);0.45,0.60,0.60) | ((7,8,9);1.00,0.0,0.0) |
|  | C_13_ | ((3,4,5);0.75,0.20,0.20) | ((6,7,8);0.9,0.10,0.10) | ((3,4,5);0.75,0.20,0.20) | ((7,8,9);1.00,0.0,0.0) | ((6,7,8);0.9,0.10,0.10) | ((3,4,5);0.75,0.20,0.20) | ((6,7,8);0.9,0.10,0.10) | ((7,8,9);1.00,0.0,0.0) | ((6,7,8);0.9,0.10,0.10) | ((3,4,5);0.75,0.20,0.20) |
|  | C_12_ | ((5,6,7);0.85,0.15,0.15) | ((7,8,9);1.00,0.0,0.0) | ((6,7,8);0.9,0.10,0.10) | ((5,6,7);0.85,0.15,0.15) | ((6,7,8);0.9,0.10,0.10) | ((7,8,9);1.00,0.0,0.0) | ((5,6,7);0.85,0.15,0.15) | ((6,7,8);0.9,0.10,0.10) | ((7,8,9);1.00,0.0,0.0) | ((5,6,7);0.85,0.15,0.15) |
|  | C_4_ | ((7,8,9);1.00,0.0,0.0) | ((5,6,7);0.85,0.15,0.15) | ((6,7,8);0.9,0.10,0.10) | ((5,6,7);0.85,0.15,0.15) | ((6,7,8);0.9,0.10,0.10) | ((7,8,9);1.00,0.0,0.0) | ((6,7,8);0.9,0.10,0.10) | ((6,7,8);0.9,0.10,0.10) | ((7,8,9);1.00,0.0,0.0) | ((5,6,7);0.85,0.15,0.15) |
|  | C_7_ | ((5,6,7);0.85,0.15,0.15) | ((7,8,9);1.00,0.0,0.0) | ((6,7,8);0.9,0.10,0.10) | ((6,7,8);0.9,0.10,0.10) | ((5,6,7);0.85,0.15,0.15) | ((7,8,9);1.00,0.0,0.0) | ((6,7,8);0.9,0.10,0.10) | ((3,4,5);0.75,0.20,0.20) | ((6,7,8);0.9,0.10,0.10) | ((7,8,9);1.00,0.0,0.0) |
|  | C_9_ | ((5,6,7);0.85,0.15,0.15) | ((6,7,8);0.9,0.10,0.10) | ((6,7,8);0.9,0.10,0.10) | ((5,6,7);0.85,0.15,0.15) | ((7,8,9);1.00,0.0,0.0) | ((6,7,8);0.9,0.10,0.10) | ((5,6,7);0.85,0.15,0.15) | ((7,8,9);1.00,0.0,0.0) | ((6,7,8);0.9,0.10,0.10) | ((5,6,7);0.85,0.15,0.15) |
| 4 | C_14_ | ((7,8,9);1.00,0.0,0.0) | ((5,6,7);0.85,0.15,0.15) | ((6,7,8);0.9,0.10,0.10) | ((5,6,7);0.85,0.15,0.15) | ((7,8,9);1.00,0.0,0.0) | ((5,6,7);0.85,0.15,0.15) | ((7,8,9);1.00,0.0,0.0) | ((5,6,7);0.85,0.15,0.15) | ((6,7,8);0.9,0.10,0.10) | ((5,6,7);0.85,0.15,0.15) |
|  | C_10_ | ((5,6,7);0.85,0.15,0.15) | ((7,8,9);1.00,0.0,0.0) | ((5,6,7);0.85,0.15,0.15) | ((6,7,8);0.9,0.10,0.10) | ((5,6,7);0.85,0.15,0.15) | ((6,7,8);0.9,0.10,0.10) | ((7,8,9);1.00,0.0,0.0) | ((7,8,9);1.00,0.0,0.0) | ((5,6,7);0.85,0.15,0.15) | ((6,7,8);0.9,0.10,0.10) |
|  | C_11_ | ((7,8,9);1.00,0.0,0.0) | ((3,4,5);0.75,0.20,0.20) | ((7,8,9);1.00,0.0,0.0) | ((3,4,5);0.75,0.20,0.20) | ((7,8,9);1.00,0.0,0.0) | ((3,4,5);0.75,0.20,0.20) | ((6,7,8);0.9,0.10,0.10) | ((7,8,9);1.00,0.0,0.0) | ((6,7,8);0.9,0.10,0.10) | ((7,8,9);1.00,0.0,0.0) |
|  | C_8_ | ((7,8,9);1.00,0.0,0.0) | ((3,4,5);0.75,0.20,0.20) | ((6,7,8);0.9,0.10,0.10) | ((7,8,9);1.00,0.0,0.0) | ((3,4,5);0.75,0.20,0.20) | ((6,7,8);0.9,0.10,0.10) | ((7,8,9);1.00,0.0,0.0) | ((3,4,5);0.75,0.20,0.20) | ((3,4,5);0.75,0.20,0.20) | ((7,8,9);1.00,0.0,0.0) |
|  | C_6_ | ((6,7,8);0.9,0.10,0.10) | ((3,4,5);0.75,0.20,0.20) | ((7,8,9);1.00,0.0,0.0) | (6,7,8);0.9,0.10,0.10) | ((6,7,8);0.9,0.10,0.10) | ((7,8,9);1.00,0.0,0.0) | ((6,7,8);0.9,0.10,0.10) | ((3,4,5);0.75,0.20,0.20) | ((7,8,9);1.00,0.0,0.0) | ((6,7,8);0.9,0.10,0.10) |
|  | C_1_ | ((7,8,9);1.00,0.0,0.0) | ((3,4,5);0.75,0.20,0.20) | ((6,7,8);0.9,0.10,0.10) | ((7,8,9);1.00,0.0,0.0) | ((6,7,8);0.9,0.10,0.10) | ((3,4,5);0.75,0.20,0.20) | ((7,8,9);1.00,0.0,0.0) | ((3,4,5);0.75,0.20,0.20) | ((6,7,8);0.9,0.10,0.10) | ((7,8,9);1.00,0.0,0.0) |
|  | C_3_ | ((6,7,8);0.9,0.10,0.10) | ((3,4,5);0.75,0.20,0.20) | ((7,8,9);1.00,0.0,0.0) | ((3,4,5);0.75,0.20,0.20) | ((6,7,8);0.9,0.10,0.10) | ((7,8,9);1.00,0.0,0.0) | ((3,4,5);0.75,0.20,0.20) | ((3,4,5);0.75,0.20,0.20) | ((7,8,9);1.00,0.0,0.0) | ((6,7,8);0.9,0.10,0.10) |
|  | C_5_ | ((7,8,9);1.00,0.0,0.0) | ((7,8,9);1.00,0.0,0.0) | ((6,7,8);0.9,0.10,0.10) | ((3,4,5);0.75,0.20,0.20) | ((7,8,9);1.00,0.0,0.0) | ((6,7,8);0.9,0.10,0.10) | ((3,4,5);0.75,0.20,0.20) | ((7,8,9);1.00,0.0,0.0) | ((6,7,8);0.9,0.10,0.10) | ((7,8,9);1.00,0.0,0.0) |
|  | C_15_ | ((3,4,5);0.75,0.20,0.20) | ((6,7,8);0.9,0.10,0.10) | ((6,7,8);0.9,0.10,0.10) | ((3,4,5);0.75,0.20,0.20) | ((6,7,8);0.9,0.10,0.10) | ((7,8,9);1.00,0.0,0.0) | ((3,4,5);0.75,0.20,0.20) | ((7,8,9);1.00,0.0,0.0) | ((7,8,9);1.00,0.0,0.0) | ((3,4,5);0.75,0.20,0.20) |
|  | C_2_ | ((7,8,9);1.00,0.0,0.0) | ((5,6,7);0.85,0.15,0.15) | ((3,4,5);0.75,0.20,0.20) | ((6,7,8);0.9,0.10,0.10) | ((7,8,9);1.00,0.0,0.0) | ((3,4,5);0.75,0.20,0.20) | ((6,7,8);0.9,0.10,0.10) | ((3,4,5);0.75,0.20,0.20) | ((6,7,8);0.9,0.10,0.10) | ((3,4,5);0.75,0.20,0.20) |
|  | C_13_ | ((3,4,5);0.75,0.20,0.20) | ((7,8,9);1.00,0.0,0.0) | ((6,7,8);0.9,0.10,0.10) | ((7,8,9);1.00,0.0,0.0) | ((3,4,5);0.75,0.20,0.20) | ((6,7,8);0.9,0.10,0.10) | ((3,4,5);0.75,0.20,0.20) | ((7,8,9);1.00,0.0,0.0) | ((3,4,5);0.75,0.20,0.20) | ((7,8,9);1.00,0.0,0.0) |
|  | C_12_ | ((7,8,9);1.00,0.0,0.0) | ((6,7,8);0.9,0.10,0.10) | ((1,2,3);0.45,0.60,0.60) | ((7,8,9);1.00,0.0,0.0) | ((6,7,8);0.9,0.10,0.10) | ((1,2,3);0.45,0.60,0.60) | ((7,8,9);1.00,0.0,0.0) | ((6,7,8);0.9,0.10,0.10) | ((7,8,9);1.00,0.0,0.0) | ((1,2,3);0.45,0.60,0.60) |
|  | C_4_ | ((3,4,5);0.75,0.20,0.20) | ((1,2,3);0.45,0.60,0.60) | ((5,6,7);0.85,0.15,0.15) | ((1,2,3);0.45,0.60,0.60) | ((5,6,7);0.85,0.15,0.15) | ((3,4,5);0.75,0.20,0.20) | ((7,8,9);1.00,0.0,0.0) | ((6,7,8);0.9,0.10,0.10) | ((1,2,3);0.45,0.60,0.60) | ((7,8,9);1.00,0.0,0.0) |
|  | C_7_ | ((5,6,7);0.85,0.15,0.15) | ((1,2,3);0.45,0.60,0.60) | ((1,2,3);0.45,0.60,0.60) | ((6,7,8);0.9,0.10,0.10) | ((7,8,9);1.00,0.0,0.0) | ((1,2,3);0.45,0.60,0.60) | ((6,7,8);0.9,0.10,0.10) | ((7,8,9);1.00,0.0,0.0) | ((5,6,7);0.85,0.15,0.15) | ((1,2,3);0.45,0.60,0.60) |
|  | C_9_ | ((1,2,3);0.45,0.60,0.60) | ((6,7,8);0.9,0.10,0.10) | ((5,6,7);0.85,0.15,0.15) | ((1,2,3);0.45,0.60,0.60) | ((6,7,8);0.9,0.10,0.10) | ((1,2,3);0.45,0.60,0.60) | ((5,6,7);0.85,0.15,0.15) | ((6,7,8);0.9,0.10,0.10) | ((1,2,3);0.45,0.60,0.60) | ((5,6,7);0.85,0.15,0.15) |
| 5 | C_14_ | ((5,6,7);0.85,0.15,0.15) | ((3,4,5);0.75,0.20,0.20) | ((6,7,8);0.9,0.10,0.10) | ((5,6,7);0.85,0.15,0.15) | ((7,8,9);1.00,0.0,0.0) | ((1,2,3);0.40,0.60,0.60) | ((3,4,5);0.75,0.20,0.20) | ((5,6,7);0.85,0.15,0.15) | ((5,6,7);0.85,0.15,0.15) | ((3,4,5);0.75,0.20,0.20) |
|  | C_10_ | ((6,7,8);0.9,0.10,0.10) | ((6,7,8);0.9,0.10,0.10) | ((5,6,7);0.85,0.15,0.15) | ((7,8,9);1.00,0.0,0.0) | ((3,4,5);0.75,0.20,0.20) | ((6,7,8);0.9,0.10,0.10) | ((5,6,7);0.85,0.15,0.15) | ((7,8,9);1.00,0.0,0.0) | ((3,4,5);0.75,0.20,0.20) | ((6,7,8);0.9,0.10,0.10) |
|  | C_11_ | ((3,4,5);0.75,0.20,0.20) | ((5,6,7);0.85,0.15,0.15) | ((6,7,8);0.9,0.10,0.10) | ((5,6,7);0.85,0.15,0.15) | ((6,7,8);0.9,0.10,0.10) | ((5,6,7);0.85,0.15,0.15) | ((3,4,5);0.75,0.20,0.20) | ((7,8,9);1.00,0.0,0.0) | ((3,4,5);0.75,0.20,0.20) | ((6,7,8);0.9,0.10,0.10) |
|  | C_8_ | ((7,8,9);1.00,0.0,0.0) | ((3,4,5);0.75,0.20,0.20) | ((5,6,7);0.85,0.15,0.15) | ((7,8,9);1.00,0.0,0.0) | ((3,4,5);0.75,0.20,0.20) | ((7,8,9);1.00,0.0,0.0) | ((5,6,7);0.85,0.15,0.15) | ((3,4,5);0.75,0.20,0.20) | ((7,8,9);1.00,0.0,0.0) | ((5,6,7);0.85,0.15,0.15) |
|  | C_6_ | ((5,6,7);0.85,0.15,0.15) | ((6,7,8);0.9,0.10,0.10) | ((3,4,5);0.75,0.20,0.20) | ((6,7,8);0.9,0.10,0.10) | ((5,6,7);0.85,0.15,0.15) | ((7,8,9);1.00,0.0,0.0) | ((6,7,8);0.9,0.10,0.10) | ((7,8,9);1.00,0.0,0.0) | ((5,6,7);0.85,0.15,0.15) | ((6,7,8);0.9,0.10,0.10) |
|  | C_1_ | ((7,8,9);1.00,0.0,0.0) | ((3,4,5);0.75,0.20,0.20) | ((6,7,8);0.9,0.10,0.10) | ((7,8,9);1.00,0.0,0.0) | ((6,7,8);0.9,0.10,0.10) | ((7,8,9);1.00,0.0,0.0) | ((6,7,8);0.9,0.10,0.10) | ((7,8,9);1.00,0.0,0.0) | ((3,4,5);0.75,0.20,0.20) | ((7,8,9);1.00,0.0,0.0) |
|  | C_3_ | ((7,8,9);1.00,0.0,0.0) | ((6,7,8);0.9,0.10,0.10) | ((3,4,5);0.75,0.20,0.20) | ((7,8,9);1.00,0.0,0.0) | ((6,7,8);0.9,0.10,0.10) | ((7,8,9);1.00,0.0,0.0) | ((3,4,5);0.75,0.20,0.20) | ((7,8,9);1.00,0.0,0.0) | ((6,7,8);0.9,0.10,0.10) | ((3,4,5);0.75,0.20,0.20) |
|  | C_5_ | ((1,2,3);0.45,0.60,0.60) | ((7,8,9);1.00,0.0,0.0) | ((6,7,8);0.9,0.10,0.10) | ((1,2,3);0.45,0.60,0.60) | ((7,8,9);1.00,0.0,0.0) | ((6,7,8);0.9,0.10,0.10) | ((7,8,9);1.00,0.0,0.0) | ((3,4,5);0.75,0.20,0.20) | ((1,2,3);0.45,0.60,0.60) | ((7,8,9);1.00,0.0,0.0) |
|  | C_15_ | ((3,4,5);0.75,0.20,0.20) | ((7,8,9);1.00,0.0,0.0) | ((3,4,5);0.75,0.20,0.20) | ((6,7,8);0.9,0.10,0.10) | ((7,8,9);1.00,0.0,0.0) | ((3,4,5);0.75,0.20,0.20) | ((3,4,5);0.75,0.20,0.20) | ((7,8,9);1.00,0.0,0.0) | ((3,4,5);0.75,0.20,0.20) | ((7,8,9);1.00,0.0,0.0) |
|  | C_2_ | ((7,8,9);1.00,0.0,0.0) | ((6,7,8);0.9,0.10,0.10) | ((3,4,5);0.75,0.20,0.20) | ((7,8,9);1.00,0.0,0.0) | ((6,7,8);0.9,0.10,0.10) | ((7,8,9);1.00,0.0,0.0) | ((3,4,5);0.75,0.20,0.20) | ((3,4,5);0.75,0.20,0.20) | ((6,7,8);0.9,0.10,0.10) | ((7,8,9);1.00,0.0,0.0) |
|  | C_13_ | ((6,7,8);0.9,0.10,0.10) | ((5,6,7);0.85,0.15,0.15) | ((7,8,9);1.00,0.0,0.0) | ((5,6,7);0.85,0.15,0.15) | ((6,7,8);0.9,0.10,0.10) | ((7,8,9);1.00,0.0,0.0) | ((6,7,8);0.9,0.10,0.10) | ((5,6,7);0.85,0.15,0.15) | ((7,8,9);1.00,0.0,0.0) | ((6,7,8);0.9,0.10,0.10) |
|  | C_12_ | ((7,8,9);1.00,0.0,0.0) | ((6,7,8);0.9,0.10,0.10) | ((5,6,7);0.85,0.15,0.15) | ((6,7,8);0.9,0.10,0.10) | ((7,8,9);1.00,0.0,0.0) | ((5,6,7);0.85,0.15,0.15) | ((6,7,8);0.9,0.10,0.10) | ((7,8,9);1.00,0.0,0.0) | ((5,6,7);0.85,0.15,0.15) | ((6,7,8);0.9,0.10,0.10) |
|  | C_4_ | ((6,7,8);0.9,0.10,0.10) | ((5,6,7);0.85,0.15,0.15) | ((6,7,8);0.9,0.10,0.10) | ((5,6,7);0.85,0.15,0.15) | ((7,8,9);1.00,0.0,0.0) | ((6,7,8);0.9,0.10,0.10) | ((7,8,9);1.00,0.0,0.0) | ((5,6,7);0.85,0.15,0.15) | ((7,8,9);1.00,0.0,0.0) | ((6,7,8);0.9,0.10,0.10) |
|  | C_7_ | ((7,8,9);1.00,0.0,0.0) | ((6,7,8);0.9,0.10,0.10) | ((5,6,7);0.85,0.15,0.15) | ((6,7,8);0.9,0.10,0.10) | ((5,6,7);0.85,0.15,0.15) | ((5,6,7);0.85,0.15,0.15) | ((6,7,8);0.9,0.10,0.10) | ((5,6,7);0.85,0.15,0.15) | ((6,7,8);0.9,0.10,0.10) | ((7,8,9);1.00,0.0,0.0) |
|  | C_9_ | ((6,7,8);0.9,0.10,0.10) | ((5,6,7);0.85,0.15,0.15) | ((6,7,8);0.9,0.10,0.10) | ((7,8,9);1.00,0.0,0.0) | ((7,8,9);1.00,0.0,0.0) | ((6,7,8);0.9,0.10,0.10) | ((5,6,7);0.85,0.15,0.15) | ((7,8,9);1.00,0.0,0.0) | ((5,6,7);0.85,0.15,0.15) | ((6,7,8);0.9,0.10,0.10) |

**Table A.3:** The combined values of alternatives according to each attribute

|  | **A_1_** | **A_2_** | **A_3_** | **A_4_** | **A_5_** | **A_6_** | **A_7_** | **A_8_** | **A_9_** | **A_10_** |
| --- | --- | --- | --- | --- | --- | --- | --- | --- | --- | --- |
| C_14_ | (27,32,37);0.75,0.20,0.20) | ((23,28,33);0.75,0.20,0.20) | ((31,36,41);0.85,0.15,0.15) | ((23,28,33);0.85,0.45,0.60,60) | ((30,35,40);0.75,0.20,0.20) | ((19,24,29);0.45,0.60,0.60) | ((28,33,38);0.75,0.20,0.20) | ((28,33,38);0.85,0.15,0.15) | ((24,29,34);0.75,0.20,0.20) | ((25,30,35);0.75,0.20,20) |
| C_10_ | ((21,26,31);0.45,0.60,0.60) | ((32,37,42);0.85,0.15,0.15) | ((22,27,32);0.75,0.20,0.20) | ((26,31,36);0.45,0.60,0.60) | ((23,28,33);0.45,0.60,0.60) | ((28,33,38);0.85,0.15,0.15) | ((27,32,37);0.75,0.20,0.20) | ((33,38,43);0.85,0.15,0.15) | ((23,28,33);0.75,0.20,0.20) | ((29,34,39);0.75,0.20,0.20) |
| C_11_ | ((21,26,31);0.45,0.60,0.60) | ((22,27,32);0.45,0.60,0.60) | ((32,37,42);0.85,0.15,0.15) | ((23,28,33);0.75,0.20,0.20) | ((28,33,38);0.75,0.20,0.20) | ((24,29,34);0.75,0.20,0.20) | ((25,30,35);0.75,0.20,0.20) | ((27,32,37);0.75,0.20,0.20) | ((26,31,36);0.75,0.20,0.20 | ((26,31,36);0.45,0.60,0.60) |
| C_8_ | ((33,38,43);0.9,0.1,0.1) | ((15,20,25);0.45,0.60,0.60) | ((25,30,35);0.75,0.20,0.20) | ((30,35,40);0.85,0.15,0.15) | ((21,26,31);0.75,0.20,0.20) | ((23,28,33);0.45,0.60,0.60) | ((24,29,34);0.45,0.60,0.60) | ((19,24,29);0.75,0.20,0.20) | ((26,31,36);0.75,0.20,0.20) | ((32,37,42);0.85,0.15,0.15) |
| C_6_ | ((26,31,36);0.75,0.20,0.20) | ((24,29,34);0.75,0.20,0.20) | ((20,25,30);0.45,0.60,0.60) | ((30,35,40);0.85,0.15,0.15) | ((20,25,30);0.75,0.20,0.20) | ((26,31,36);0.45,0.60,0.60) | ((22,27,32);0.45,0.60,0.60) | ((25,30,35);0.75,0.20,0.20) | ((28,33,38);0.75,0.20,0.20) | ((23,28,33);0.75,0.20,0.20) |
| C_1_ | ((26,31,36);0.45,0.60,0.60) | ((21,26,31);0.75,0.20,0.20) | ((25,30,35);0.45,0.60,0.60) | ((27,32,37);0.75,0.20,0.20) | ((19,24,29);0.45,0.60,0.60) | ((22,27,32);0.75,0.20,0.20) | ((31,36,41);0.85,0.15,0.15) | ((20,25,30);0.45,0.60,0.60) | ((20,25,30);0.75,0.20,0.20) | ((22,27,32);0.45,0.60,0.60) |
| C_3_ | ((22,27,32);0.45,0.60,0.60) | ((22,27,32);0.75,0.20,0.20) | ((23,28,33);0.75,0.20,0.20) | ((27,32,37)0.75,0.20,0.20) | ((23,28,33);0.45,0.60,0.60) | ((26,31,36);0.75,0.20,0.20) | ((15,20,25);0.45,0.60,0.60 | ((25,30,35);0.75,0.20,0.20) | ((30,35,40);0.85,0.15,0.15) | ((18,23,28);0.45,0.60,0.60) |
| C_5_ | ((23,28,33);0.45,0.60,0.60) | ((26,31,36);0.75,0.20,0.20) | ((28,33,38);0.75,0.20,0.20) | ((18,23,28);0.45,0.60,0.60) | ((31,36,41);0.75,0.20,0.20) | ((27,32,37);0.75,0.20,0.20) | ((24,29,34);0.45,0.60,0.60) | ((23,28,33);0.75,0.20,0.20) | ((22,27,32);0.45,0.60,0.60) | ((30,35,40);0.75,0.20,0.20) |
| C_15_ | ((19,24,29);0.45,0.60,0.60) | ((33,38,43);0.9,0.1,0.1) | ((24,29,34);0.75,0.20,0.20) | ((26,31,36);0.75,0.20,0.20) | ((29,34,39);0.85,0.15,0.15) | ((23,28,33);0.45,0.60,0.60) | ((23,28,33);0.75,0.20,0.20) | ((27,32,37);0.75,0.20,0.20) | ((22,27,32);0.45,0.60,0.60) | ((24,29,34);0.45,0.60,0.60) |
| C_2_ | ((34,39,44);0.9,0.1,0.1) | ((27,32,37);0.75,0.20,0.20) | ((23,28,33);0.75,0.20,0.20) | ((24,29,34);0.45,0.60,0.60) | ((24,29,34);0.75,0.20,0.20) | ((25,30,35);0.45,0.60,0.60) | ((21,26,31);0.45,0.60,0.60) | ((17,22,27);0.45,0.60,0.60) | ((20,25,30);0.45,0.60,0.60) | ((25,30,35);0.75,0.20,0.20) |
| C_13_ | ((16,21,26);0.45,0.60,0.60) | ((30,35,40);0.85,0.15,0.15) | ((22,27,32);0.45,0.60,0.60) | ((31,36,41);0.85,0.15,0.15) | ((23,28,33);0.45,0.60,0.60) | ((24,29,34);0.75,0.20,0.20) | ((26,31,36);0.75,0.20,0.20) | ((29,34,39);0.85,0.15,0.15) | ((24,29,34);0.45,0.60,0.60) | ((30,35,40);0.75,0.20,0.20) |
| C_12_ | ((29,34,39);0.75,0.20,0.20) | ((31,36,41);0.85,0.15,0.15) | ((24,29,34,);0.45,0.60,0.60) | ((26,31,36);0.75,0.20,0.20) | ((33,38,43);0.9,0.1,0.1) | ((26,31,36);0.45,0.60,0.60) | ((28,33,38,);0.75,0.20,0.20) | ((30,35,40);0.85,0.15,0.15) | ((23,28,33);0.45,0.60,0.60) | ((20,25,30);0.45,0.60,0.60) |
| C_4_ | ((26,31,36);0.75,0.20,0.20) | ((21,26,31);0.45,0.60,0.60) | ((25,30,35);0.75,0.20,0.20) | ((23,28,33);0.45,0.60,0.60) | ((29,34,39);0.85,0.15,0.15) | ((20,25,30);0.45,0.60,0.60) | ((33,38,43);0.9,0.10,0.10) | ((19,24,29);0.45,0.60,0.60) | ((27,32,37);0.45,0.60,0.60) | ((28,33,38);0.75,0.20,0.20) |
| C_7_ | ((25,30,35);0.75,0.20,0.20) | ((24,29,34);0.45,0.60,0.60) | ((21,26,31);0.45,0.60,0.60) | ((28,33,38);0.75,0.20,0.20) | ((28,33,38);0.85,0.15,0.15) | ((26,31,36);0.45,0.60,0.60) | ((29,34,39);0.85,0.15,0.15) | ((19,24,29);0.45,0.60,0.60) | ((27,32,37);0.85,0.15,0.15) | ((29,34,39);0.45,0.60,0.60) |
| C_9_ | ((22,27,32);0.45,0.60,0.60) | ((26,31,36);0.75,0.20,0.20) | ((27,32,37)0.75,0.20,0.20) | ((24,29,34);0.45,0.60,0.60) | ((30,35,40);0.75,0.20,0.20) | ((21,26,31);0.45,0.60,0.60) | ((28,33,38);0.85,0.15,0.15) | ((34,39,44);0.9,0.10,0.10) | ((25,30,35);0.45,0.60,0.60) | ((29,34,39);0.85,0.15,0.15) |

**Appendix B**

The model of OPA_N is as follow:

Max= z

S.t:

$$1*(1*(w_{c_{14A_{3}}}- w_{c_{14A_{8}}} ))\geq z$$

$$1*(2*(w_{c_{14A_{8}}}- w_{c_{14A_{5}}} )) \geq z$$

$$1*(3*(w_{c_{14A_{5}}}- w_{c_{14A_{7}}} )) \geq z$$

$1*(4*(w_{c_{14A_{7}}}- w_{c_{14A_{1}}})) \geq z$ (13)

$$1*(5*(w_{c_{14A_{1}}}- w_{c_{14A_{10}}} )) \geq z$$

$$1*(6*(w_{c_{14A_{10}}}- w_{c_{14A_{9}}} )) \geq z$$

$$1*(7*(w_{c_{14A_{9}}}- w_{c_{14A_{2}}} )) \geq z$$

$$1*(8*(w_{c_{14A_{2}}}- w_{c_{14A_{4}}} )) \geq z$$

$$1*(9*(w_{c_{14A_{4}}}- w_{c_{14A_{6}}} )) \geq z$$

$$1*(10*(w_{c_{14A_{6}}} )) \geq z$$

$$2*(1*(w_{c_{10A_{8}}}- w_{c_{10A_{2}}} ))\geq z$$

$$2*(2*(w_{c_{10A_{2}}}- w_{c_{10A_{6}}} )) \geq z$$

$$2*(3*(w_{c_{10A_{6}}}- w_{c_{10A_{10}}} )) \geq z$$

$$2*(4*(w_{c_{10A_{10}}}- w_{c_{10A_{7}}} )) \geq z$$

$$2*(5*(w_{c_{10A_{7}}}- w_{c_{10A_{9}}} )) \geq z$$

$$2*(6*(w_{c_{10A_{9}}}- w_{c_{10A_{3}}} )) \geq z$$

$$2*(7*(w_{c_{10A_{3}}}- w_{c_{10A_{4}}} )) \geq z$$

$$2*(8*(w_{c_{10A_{4}}}- w_{c_{10A_{5}}} )) \geq z$$

$$2*(9*(w_{c_{10A_{5}}}- w_{c_{10A_{1}}})) \geq z$$

$$2*(10*(w_{c_{10A_{1}}})) \geq z$$

$$3*(1*(w_{c_{11A_{3}}}- w_{c_{11A_{5}}} ))\geq z$$

$$3*(2*(w_{c_{11A_{5}}}- w_{c_{11A_{8}}} )) \geq z$$

$$3*(3*(w_{c_{11A_{8}}}- w_{c_{11A_{9}}} )) \geq z$$

$$3*(4*(w_{c_{11A_{9}}}- w_{c_{11A_{7}}} )) \geq z$$

$$3*(5*(w_{c_{11A_{7}}}- w_{c_{11A_{6}}} )) \geq z$$

$$3*(6*(w_{c_{11A_{6}}}- w_{c_{11A_{4}}} )) \geq z$$

$$3*(7*(w_{c_{11A_{4}}}- w_{c_{11A_{10}}} )) \geq z$$

$$3*(8*(w_{c_{11A_{10}}}- w_{c_{11A_{2}}} )) \geq z$$

$$3*(9*(w_{c_{11A_{2}}}- w_{c_{11A_{1}}})) \geq z$$

$$3*(10*(w_{c_{11A_{1}}})) \geq z$$

$$4*(1*(w_{c_{8A_{1}}}- w_{c_{8A_{10}}} ))\geq z$$

$$4*(2*(w_{c_{8A_{10}}}- w_{c_{8A_{4}}} )) \geq z$$

$$4*(3*(w_{c_{8A_{4}}}- w_{c_{8A_{9}}} )) \geq z$$

$$4*(4*(w_{c_{8A_{9}}}- w_{c_{8A_{3}}} )) \geq z$$

$$4*(5*(w_{c_{8A_{3}}}- w_{c_{8A_{5}}} )) \geq z$$

$$4*(6*(w_{c_{8A_{5}}}- w_{c_{8A_{8}}} )) \geq z$$

$$4*(7*(w_{c_{8A_{8}}}- w_{c_{8A_{7}}} )) \geq z$$

$$4*(8*(w_{c_{8A_{7}}}- w_{c_{8A_{6}}} )) \geq z$$

$$4*(9*(w_{c_{8A_{6}}}- w_{c_{8A_{2}}})) \geq z$$

$$4*(10*(w_{c_{8A_{2}}})) \geq z$$

$$5*(1*(w_{c_{6A_{4}}}- w_{c_{6A_{9}}}))\geq z$$

$$5*(2*(w_{c_{6A_{9}}}- w_{c_{6A_{1}}})) \geq z$$

$$5*(3*(w_{c_{6A_{1}}}- w_{c_{6A_{8}}} )) \geq z$$

$$5*(4*(w_{c_{6A_{8}}}- w_{c_{6A_{2}}} )) \geq z$$

$$5*(5*(w_{c_{6A_{2}}}- w_{c_{6A_{10}}} )) \geq z$$

$$5*(6*(w_{c_{6A_{10}}}- w_{c_{6A_{5}}} )) \geq z$$

$$5*(7*(w_{c_{6A_{5}}}- w_{c_{6A_{6}}} )) \geq z$$

$$5*(8*(w_{c_{6A_{6}}}- w_{c_{6A_{7}}} )) \geq z$$

$$5*(9*(w_{c_{6A_{7}}}- w_{c_{6A_{3}}} )) \geq z$$

$$5*(10*(w_{c_{6A_{3}}} )) \geq z$$

$$6*(1*(w_{c_{1A_{7}}}- w_{c_{1A_{4}}} ))\geq z$$

$$6*(2*(w_{c_{1A_{4}}}- w_{c_{1A_{6}}} )) \geq z$$

$$6*(3*(w_{c_{1A_{6}}}- w_{c_{1A_{2}}} )) \geq z$$

$$6*(4*(w_{c_{1A_{2}}}- w_{c_{1A_{9}}} )) \geq z$$

$$6*(5*(w_{c_{1A_{9}}}- w_{c_{1A_{1}}})) \geq z$$

$$6*(6*(w_{c_{1A_{1}}}- w_{c_{1A_{3}}} )) \geq z$$

$$6*(7*(w_{c_{1A_{3}}}- w_{c_{1A_{10}}} )) \geq z$$

$$6*(8*(w_{c_{1A_{10}}}- w_{c_{1A_{8}}} )) \geq z$$

$$6*(9*(w_{c_{1A_{8}}}- w_{c_{1A_{5}}} )) \geq z$$

$$6*(10*(w_{c_{1A_{5}}})) \geq z$$

$$7*(1*(w_{c_{3A_{9}}}- w_{c_{3A_{4}}}))\geq z$$

$$7*(2*(w_{c_{3A_{4}}}- w_{c_{3A_{6}}})) \geq z$$

$$7*(3*(w_{c_{3A_{6}}}- w_{c_{3A_{8}}} )) \geq z$$

$$7*(4*(w_{c_{3A_{8}}}- w_{c_{3A_{3}}} )) \geq z$$

$$7*(5*(w_{c_{3A_{3}}}- w_{c_{3A_{2}}} )) \geq z$$

$$7*(6*(w_{c_{3A_{2}}}- w_{c_{3A_{5}}} )) \geq z$$

$$7*(7*(w_{c_{3A_{5}}}- w_{c_{3A_{1}}})) \geq z$$

$$7*(8*(w_{c_{3A_{1}}}- w_{c_{3A_{10}}} )) \geq z$$

$$7*(9*(w_{c_{3A_{10}}}- w_{c_{3A_{7}}} )) \geq z$$

$$7*(10*(w_{c_{3A_{7}}}))) \geq z$$

$$8*(1*(w_{c_{5A_{5}}}- w_{c_{5A_{10}}} ))\geq z$$

$$8*(2*(w_{c_{5A_{10}}}- w_{c_{5A_{3}}} )) \geq z$$

$$8*(3*(w_{c_{5A_{3}}}- w_{c_{5A_{6}}} )) \geq z$$

$$8*(4*(w_{c_{5A_{6}}}- w_{c_{5A_{2}}} )) \geq z$$

$$8*(5*(w_{c_{5A_{2}}}- w_{c_{5A_{8}}} )) \geq z$$

$$8*(6*(w_{c_{5A_{8}}}- w_{c_{5A_{7}}} )) \geq z$$

$$8*(7*(w_{c_{5A_{7}}}- w_{c_{5A_{1}}})) \geq z$$

$$8*(8*(w_{c_{5A_{1}}}- w_{c_{5A_{9}}} )) \geq z$$

$$8*(9*(w_{c_{5A_{9}}}- w_{c_{5A_{4}}} )) \geq z$$

$$8*(10*(w_{c_{5A_{4}}})) \geq z$$

$$9*(1*(w_{c_{15A_{2}}}- w_{c_{15A_{5}}}))\geq z$$

$$9*(2*(w_{c_{15A_{5}}}- w_{c_{15A_{8}}} )) \geq z$$

$$9*(3*(w_{c_{15A_{8}}}- w_{c_{15A_{4}}} )) \geq z$$

$$9*(4*(w_{c_{15A_{4}}}- w_{c_{15A_{3}}} )) \geq z$$

$$9*(5*(w_{c_{15A_{3}}}- w_{c_{15A_{7}}} )) \geq z$$

$$9*(6*(w_{c_{15A_{7}}}- w_{c_{15A_{10}}} )) \geq z$$

$$9*(7*(w_{c_{15A_{10}}}- w_{c_{15A_{6}}} )) \geq z$$

$$9*(8*(w_{c_{15A_{6}}}- w_{c_{15A_{9}}} )) \geq z$$

$$9*(9*(w_{c_{15A_{9}}}- w_{c_{15A_{1}}})) \geq z$$

$$9*(10*(w_{c_{15A_{1}}})) \geq z$$

$$10*(1*(w_{c_{2A_{1}}}- w_{c_{2A_{2}}} ))\geq z$$

$$10*(2*(w_{c_{2A_{2}}}- w_{c_{2A_{10}}} )) \geq z$$

$$10*(3*(w_{c_{2A_{10}}}- w_{c_{2A_{5}}} )) \geq z$$

$$10*(4*(w_{c_{2A_{5}}}- w_{c_{2A_{3}}} )) \geq z$$

$$10*(5*(w_{c_{2A_{3}}}- w_{c_{2A_{6}}})) \geq z$$

$$10*(6*(w_{c_{2A_{6}}}- w_{c_{2A_{4}}})) \geq z$$

$$10*(7*(w_{c_{2A_{4}}}- w_{c_{2A_{7}}} )) \geq z$$

$$10*(8*(w_{c_{2A_{7}}}- w_{c_{2A_{9}}} )) \geq z$$

$$10*(9*(w_{c_{2A_{9}}}- w_{c_{2A_{8}}} )) \geq z$$

$$10*(10*(w_{c_{2A_{8}}})) \geq z$$

$$11*(1*(w_{c_{13A_{4}}}- w_{c_{13A_{2}}}))\geq z$$

$$11*(2*(w_{c_{13A_{2}}}- w_{c_{13A_{8}}} )) \geq z$$

$$11*(3*(w_{c_{13A_{8}}}- w_{c_{13A_{10}}} )) \geq z$$

$$11*(4*(w_{c_{13A_{10}}}- w_{c_{13A_{7}}} )) \geq z$$

$$11*(5*(w_{c_{13A_{7}}}- w_{c_{13A_{6}}} )) \geq z$$

$$11*(6*(w_{c_{13A_{6}}}- w_{c_{13A_{9}}} )) \geq z$$

$$11*(7*(w_{c_{13A_{9}}}- w_{c_{13A_{5}}} )) \geq z$$

$$11*(8*(w_{c_{13A_{5}}}- w_{c_{13A_{3}}} )) \geq z$$

$$11*(9*(w_{c_{13A_{3}}}- w_{c_{13A_{1}}})) \geq z$$

$$11*(10*(w_{c_{13A_{1}}})) \geq z$$

$$12*(1*(w_{c_{12A_{5}}}- w_{c_{12A_{2}}} ))\geq z$$

$$12*(2*(w_{c_{12A_{2}}}- w_{c_{12A_{8}}} )) \geq z$$

$$12*(3*(w_{c_{12A_{8}}}- w_{c_{12A_{1}}})) \geq z$$

$$12*(4*(w_{c_{12A_{1}}}- w_{c_{12A_{7}}} )) \geq z$$

$$12*(5*(w_{c_{12A_{7}}}- w_{c_{12A_{4}}} )) \geq z$$

$$12*(6*(w_{c_{12A_{4}}}- w_{c_{12A_{6}}} )) \geq z$$

$$12*(7*(w_{c_{12A_{6}}}- w_{c_{12A_{3}}} )) \geq z$$

$$12*(8*(w_{c_{12A_{3}}}- w_{c_{12A_{9}}} )) \geq z$$

$$12*(9*(w_{c_{12A_{9}}}- w_{c_{12A_{10}}} )) \geq z$$

$$12*(10*(w_{c_{12A_{10}}} )) \geq z$$

$$13*(1*(w_{c_{4A_{7}}}- w_{c_{4A_{5}}}))\geq z$$

$$13*(2*(w_{c_{4A_{5}}}- w_{c_{4A_{10}}} )) \geq z$$

$$13*(3*(w_{c_{4A_{10}}}- w_{c_{4A_{1}}})) \geq z$$

$$13*(4*(w_{c_{4A_{1}}}- w_{c_{4A_{3}}} )) \geq z$$

$$13*(5*(w_{c_{4A_{3}}}- w_{c_{4A_{9}}} )) \geq z$$

$$13*(6*(w_{c_{4A_{9}}}- w_{c_{4A_{4}}} )) \geq z$$

$$13*(7*(w_{c_{4A_{4}}}- w_{c_{4A_{2}}} )) \geq z$$

$$13*(8*(w_{c_{4A_{2}}}- w_{c_{4A_{6}}} )) \geq z$$

$$13*(9*(w_{c_{4A_{6}}}- w_{c_{4A_{8}}} )) \geq z$$

$$13*(10*(w_{c_{4A_{8}}} )) \geq z$$

$$14*(1*(w_{c_{7A_{7}}}- w_{c_{7A_{5}}} ))\geq z$$

$$14*(2*(w_{c_{7A_{5}}}- w_{c_{7A_{9}}} )) \geq z$$

$$14*(3*(w_{c_{7A_{9}}}- w_{c_{7A_{4}}} )) \geq z$$

$$14*(4*(w_{c_{7A_{4}}}- w_{c_{7A_{1}}})) \geq z$$

$$14*(5*(w_{c_{7A_{1}}}- w_{c_{7A_{10}}} )) \geq z$$

$$14*(6*(w_{c_{7A_{10}}}- w_{c_{7A_{6}}}) )) \geq z$$

$$14*(7*(w_{c_{7A_{6}}}- w_{c_{7A_{2}}} )) \geq z$$

$$14*(8*(w_{c_{7A_{2}}}- w_{c_{7A_{3}}}) )) \geq z$$

$$14*(9*(w_{c_{7A_{3}}}- w_{c_{7A_{8}}}) )) \geq z$$

$$14*(10*(w_{c_{7A_{8}}}) )) \geq z$$

$$15*(1*(w_{c_{9A_{8}}}- w_{c_{9A_{10}}} ))\geq z$$

$$15*(2*(w_{c_{9A_{10}}}- w_{c_{9A_{7}}} )) \geq z$$

$$15*(3*(w_{c_{9A_{7}}}- w_{c_{9A_{5}}} )) \geq z$$

$$15*(4*(w_{c_{9A_{5}}}- w_{c_{9A_{3}}} )) \geq z$$

$$15*(5*(w_{c_{9A_{3}}}- w_{c_{9A_{2}}} )) \geq z$$

$$15*(6*(w_{c_{9A_{2}}}- w_{c_{9A_{9}}} )) \geq z$$

$$15*(7*(w_{c_{9A_{9}}}- w_{c_{9A_{4}}} )) \geq z$$

$$15*(8*(w_{c_{9A_{4}}}- w_{c_{9A_{1}}})) \geq z$$

$$15*(9*(w_{c_{9A_{1}}}- w_{c_{9A_{6}}} )) \geq z$$

$$15*(10*(w_{c_{9A_{6}}})) \geq z$$

$$w_{c_{14A_{1}}}+ w_{c_{14A_{2}}}+ w_{c_{14A_{3}}}+ w_{c_{14A_{4}}}+ w_{c_{14A_{5}}}+ w_{c_{14A_{6}}}+( w_{c_{14A_{7}}}+ w_{c_{14A_{8}}}+ w_{c_{14A_{9}}}$$

$$+ w_{c_{14A_{10}}}+ w_{c_{10A_{1}}}+ w_{c_{10A_{2}}}+ w_{c_{10A_{3}}}+ w_{c_{10A_{4}}}+ w_{c_{10A_{5}}}+ w_{c_{10A_{6}}}+ w_{c_{10A_{7}}}+ w_{c_{10A_{8}}}$$

$+ w_{c_{10A_{9}}}+ w_{c_{10A_{10}}}+w_{c_{11A_{1}}}+ w_{c_{11A_{2}}}+w_{c_{11A_{3}}}+ w_{c_{11A_{4}}}+ w_{c_{11A_{5}}}+ w_{c_{11A_{6}}}+ w_{c_{11A_{7}}}+w_{c_{11A_{8}}} +$ $w_{c_{11A_{9}}}+ w_{c_{11A_{10}}}+ w_{c_{8A_{1}}}+ w_{c_{8A_{2}}}+ w_{c_{8A_{3}}}+ w_{c_{8A_{4}}}+ w_{c_{8A_{5}}}+ w_{c_{8A_{6}}}$+ $w_{c_{8A_{7}}}+ w_{c_{8A_{8}}}+ w_{c_{8A_{9}}}+ w_{c_{8A_{10}}}+w_{c_{6A_{1}}}+w_{c_{6A_{2}}}+ w_{c_{6A_{3}}}+ w_{c_{6A_{4}}}+ w_{c_{6A_{5}}} +w_{c_{6A_{6}}} +w_{c_{6A_{7}}}+ w_{c_{6A_{8}}}+ w_{c_{6A_{9}}}+ w_{c_{6A_{10}}}+ w_{c_{1A_{1}}}+ w_{c_{1A_{2}}}+ w_{c_{1A_{3}}}+ w_{c_{1A_{4}}}+ w_{c_{1A_{5}}}+ w_{c_{1A_{6}}}+w_{c_{1A_{7}}}+ w_{c_{1A_{8}}}+ w_{c_{1A_{9}}}+ w_{c_{1A_{10}}}+w_{c_{3A_{1}}}+ w_{c_{3A_{2}}}+ w_{c_{3A_{3}}}+ w_{c_{3A_{4}}}+ w_{c_{3A_{5}}}+ w_{c_{3A_{6}}}+ w_{c_{3A_{7}}}+ w_{c_{3A_{8}}}+ w_{c_{3A_{9}}}+ w_{c_{3A_{10}}}+w_{c_{5A_{1}}}+ w_{c_{5A_{2}}}+ w_{c_{5A_{3}}}+ w_{c_{5A_{4}}}+ w_{c_{5A_{5}}}+ w_{c_{5A_{6}}}+ w_{c_{5A_{7}}}+ w_{c_{5A_{8}}}+ w_{c_{5A_{9}}}+ w_{c_{5A_{10}}}+w_{c_{15A_{1}}}+ w_{c_{15A_{2}}}+ w_{c_{15A_{3}}}+ w_{c_{15A_{4}}}+ w_{c_{15A_{5}}}+ w_{c_{15A_{6}}}+ w_{c_{15A_{7}}}+ w_{c_{15A_{8}}}+ w_{c_{15A_{9}}}$ $+ w_{c_{15A_{10}}}+ w_{c_{2A_{1}}}+ w_{c_{2A_{2}}}+ w_{c_{2A_{3}}}+ w_{c_{2A_{4}}}+ w_{c_{2A_{5}}}+ w_{c_{2A_{6}}} +w_{c_{2A_{7}}}+ w_{c_{2A_{8}}}+ w_{c_{2A_{9}}}+ w_{c_{2A_{10}}}+ w_{c_{13A_{1}}}+ w_{c_{13A_{2}}}+ w_{c_{13A_{3}}}+ w_{c_{13A_{4}}}+ w_{c_{13A_{5}}} +w_{c_{13A_{6}}}+ w_{c_{13A_{7}}}+ w_{c_{13A_{8}}}+ w_{c_{13A_{9}}}+ w_{c_{13A_{10}}}+ w_{c_{12A_{1}}}+ w_{c_{12A_{2}}}+ w_{c_{12A_{3}}}+ w_{c_{12A_{4}}}+ w_{c_{12A_{5}}}+$ $w_{c_{12A_{6}}}+ w_{c_{12A_{7}}}+ w_{c_{12A_{8}}}+ w_{c_{12A_{9}}}+ w_{c_{12A_{10}}}+$ $w_{c_{4A_{1}}}+ w_{c_{4A_{2}}}+ w_{c_{4A_{3}}}+ w_{c_{4A_{4}}}+ w_{c_{4A_{5}}}+ w_{c_{4A_{6}}}+ w_{c_{4A_{7}}}+ w_{c_{4A_{8}}}+ w_{c_{4A_{9}}}+ w_{c_{4A_{10}}}+ w_{c_{7A_{1}}}+ w_{c_{7A_{2}}}+ w_{c_{7A_{3}}}+ w_{c_{7A_{4}}}+ w_{c_{7A_{5}}}+ w_{c_{7A_{6}}}+ w_{c_{7A_{7}}}+ w_{c_{7A_{8}}}+ w_{c_{7A_{9}}}+ w_{c_{7A_{10}}} +w_{c_{9A_{1}}}+ w_{c_{9A_{2}}}+ w_{c_{9A_{3}}}+ w_{c_{9A_{4}}}+ w_{c_{9A_{5}}}+ w_{c_{9A_{6}}}+ w_{c_{9A_{7}}}+ w_{c_{9A_{8}}}+ w_{c_{9A_{9}}}+ w_{c_{9A_{10}}}=1$

$$w_{c_{14A1}},w_{c_{14A_{2}}}, w_{c_{14A_{3}}}, w_{c_{14A_{4}}}, w_{c_{14A_{5}}}, w_{c_{14A_{6}}}, w_{c_{14A_{7}}}, w_{c_{14A_{8}}}, w_{c_{14A_{9}}}, w_{c_{14A_{10}}},w_{c_{10A_{1}}}, w_{c_{10A_{2}}}, w_{c_{10A_{3}}}, w_{c_{10A_{4}}},$$

$$w_{c_{10A_{5}}}, w_{c_{10A_{6}}}, w_{c_{10A_{7}}}, w_{c_{10A_{8}}}, w_{c_{10A_{9}}}, w_{c_{10A_{10}}},w_{c_{11A_{1}}}, w_{c_{11A_{2}}}, w_{c_{11A_{3}}}, w_{c_{11A_{4}}}, w_{c_{11A_{5}}}, w_{c_{11A_{6}}}, w_{c_{11A_{7}}}, w_{c_{11A_{8}}},$$

$$w_{c_{11A_{9}}}, w_{c_{11A_{10}}}, w_{c_{8A_{1}}}, w_{c_{8A_{2}}}, w_{c_{8A_{3}}}, w_{c_{8A_{4}}}, w_{c_{8A_{5}}}, w_{c_{8A_{6}}}, w_{c_{8A_{7}}}, w_{c_{8A_{8}}}, w_{c_{8A_{9}}}, w_{c_{8A_{10}}},w_{c_{6A_{1}}}, w_{c_{6A_{2}}}, w_{c_{6A_{3}}}, w_{c_{6A_{4}}},$$

$$w_{c_{6A_{5}}}, w_{c_{6A_{6}}}, w_{c_{6A_{7}}}, w_{c_{6A_{8}}}, w_{c_{6A_{9}}}, w_{c_{6A_{10}}} ,w_{c_{1A1}},w_{c_{1A_{2}}}, w_{c_{1A_{3}}}, w_{c_{1A_{4}}}, w_{c_{1A_{5}}}, w_{c_{1A_{6}}}, w_{c_{1A_{7}}}, w_{c_{1A_{8}}}, w_{c_{1A_{9}}}, w_{c_{1A_{10}}} ,$$

$$w_{c_{3A1}},w_{c_{3A_{2}}}, w_{c_{3A_{3}}}, w_{c_{3A_{4}}}, w_{c_{3A_{5}}}, w_{c_{3A_{6}}}, w_{c_{3A_{7}}}, w_{c_{3A_{8}}}, w_{c_{3A_{9}}}, w_{c_{3A_{10}}},w_{c_{5A1}},w_{c_{5A_{2}}}, w_{c_{5A_{3}}}, w_{c_{5A_{4}}}, w_{c_{5A_{5}}}, w_{c_{5A_{6}}},$$

$$w_{c_{5A_{7}}}, w_{c_{5A_{8}}}, w_{c_{5A_{9}}}, w_{c_{5A_{10}}} ,w_{c_{15A1}},w_{c_{15A_{2}}}, w_{c_{15A_{3}}}, w_{c_{15A_{4}}}, w_{c_{15A_{5}}}, w_{c_{15A_{6}}}, w_{c_{15A_{7}}}, w_{c_{15A_{8}}}, w_{c_{15A_{9}}}, w_{c_{15A_{10}}} ,$$

$$w_{c_{2A1}},w_{c_{2A_{2}}}, w_{c_{2A_{3}}}, w_{c_{2A_{4}}}, w_{c_{2A_{5}}}, w_{c_{2A_{6}}}, w_{c_{2A_{7}}}, w_{c_{2A_{8}}}, w_{c_{2A_{9}}}, w_{c_{2A_{10}}} ,w_{c_{13A1}},w_{c_{13A_{2}}}, w_{c_{13A_{3}}}, w_{c_{13A_{4}}}, w_{c_{13A_{5}}},$$

$$w_{c_{13A_{6}}}, w_{c_{13A_{7}}}, w_{c_{13A_{8}}}, w_{c_{13A_{9}}}, w_{c_{13A_{10}}} ,w_{c_{12A1}},w_{c_{12A_{2}}}, w_{c_{12A_{3}}}, w_{c_{12A_{4}}}, w_{c_{12A_{5}}}, w_{c_{12A_{6}}}, w_{c_{12A_{7}}} , w_{c_{12A_{8}}}, w_{c_{12A_{9}}},$$

$$w_{c_{12A_{10}}} ,w_{c_{4A1}},w_{c_{4A_{2}}}, w_{c_{4A_{3}}}, w_{c_{4A_{4}}}, w_{c_{4A_{5}}}, w_{c_{4A_{6}}}, w_{c_{4A_{7}}} , w_{c_{4A_{8}}}, w_{c_{4A_{9}}}, w_{c_{4A_{10}}},w_{c_{7A1}},w_{c_{7A_{2}}}, w_{c_{7A_{3}}}, w_{c_{7A_{4}}}, w_{c_{7A_{5}}},$$

$$w_{c_{7A_{6}}}, w_{c_{7A_{7}}}, w_{c_{7A_{8}}}, w_{c_{7A_{9}}}, w_{c_{7A_{10}}},w_{c_{9A1}},w_{c_{9A_{2}}}, w_{c_{9A_{3}}}, w_{c_{9A_{4}}}, w_{c_{9A_{5}}}, w_{c_{9A_{6}}}, w_{c_{9A_{7}}}, w_{c_{9A_{8}}}, w_{c_{9A_{9}}}, w_{c_{9A_{10}}}\geq0$$

$$w_{c_{14}}=w_{c_{14A_{1}}}+w_{c_{14A_{2}}}+w_{c_{14A_{3}}}+w_{c_{14A_{4}}}+w_{c_{14A_{5}}}+w_{c_{14A_{6}}}+w_{c_{14A_{7}}}+w_{c_{14A_{8}}}+w_{c_{14A_{9}}}$$

$$+w_{c_{14A_{10}}}$$

$$w_{c_{10}}=w_{c_{10A_{1}}}+w_{c_{10A_{2}}}+w_{c_{10A_{3}}}+w_{c_{10A_{4}}}+w_{c_{10A_{5}}}+w_{c_{10A_{6}}}+w_{c_{10A_{7}}}+w_{c_{10A_{8}}}+w_{c_{10A_{9}}}$$

$$+w_{c_{10A_{10}}}$$

$$w_{c_{11}}=w_{c_{11A_{1}}}+w_{c_{11A_{2}}}+w_{c_{11A_{3}}}+w_{c_{11A_{4}}}+w_{c_{11A_{5}}}+w_{c_{11A_{6}}}+w_{c_{11A_{7}}}+w_{c_{11A_{8}}}+w_{c_{11A_{9}}}$$

$$+w_{c_{11A_{10}}}$$

$$w_{c_{8}}=w_{c_{8A1}}+w_{c_{8A_{2}}}+w_{c_{8A_{3}}}+w_{c_{8A_{4}}}+ w_{c_{8A_{5}}}+w_{c_{8A_{6}}}+w_{c_{8A_{7}}}+w_{c_{8A_{8}}}+w_{c_{8A_{9}}}+w_{c_{8A_{10}}}$$

$$w_{c_{6}}=w_{c_{6A_{1}}}+w_{c_{6A_{2}}}+w_{c_{6A_{3}}}+w_{c_{6A_{4}}}+w_{c_{6A_{5}}}+w_{c_{6A_{6}}}+w_{c_{6A_{7}}}+w_{c_{6A_{8}}}+w_{c_{6A_{9}}}+w_{c_{6A_{10}}}$$

$$w_{c_{1}}=w_{c_{1A_{1}}}+w_{c_{1A_{2}}}+w_{c_{1A_{3}}}+w_{c_{1A_{4}}}+w_{c_{1A_{5}}}+w_{c_{1A_{6}}}+w_{c_{1A_{7}}}+w_{c_{1A_{8}}}+w_{c_{1A_{9}}}+w_{c_{1A_{10}}}$$

$$w_{c_{3}}=w_{c_{3A_{1}}}+w_{c_{3A_{2}}}+w_{c_{3A_{3}}}+w_{c_{3A_{4}}}+w_{c_{3A_{5}}}+w_{c_{3A_{6}}}+w_{c_{3A_{7}}}+w_{c_{3A_{8}}}+w_{c_{3A_{9}}}+w_{c_{3A_{10}}}$$

$$w_{c_{5}}=w_{c_{5A_{1}}}+w_{c_{5A_{2}}}+w_{c_{5A_{3}}}+w_{c_{5A_{4}}}+w_{c_{5A_{5}}}+w_{c_{5A_{6}}}+w_{c_{5A_{7}}}+w_{c_{5A_{8}}}+w_{c_{5A_{9}}}+w_{c_{5A_{10}}}$$

$$w_{c_{15}}=w_{c_{15A_{1}}}+w_{c_{15A_{2}}}+w_{c_{15A_{3}}}+w_{c_{15A_{4}}}+w_{c_{15A_{5}}}+w_{c_{15A_{6}}}+w_{c_{15A_{7}}}+w_{c_{15A_{8}}}+w_{c_{15A_{9}}}$$

$$+w_{c_{15A_{10}}}$$

$$w_{c_{2}}=w_{c_{2A1}}+w_{c_{2A_{2}}}+w_{c_{2A_{3}}}+w_{c_{2A_{4}}}+ w_{c_{2A_{5}}}+w_{c_{2A_{6}}}+w_{c_{2A_{7}}}+w_{c_{2A_{8}}}+w_{c_{2A_{9}}}+w_{c_{2A_{10}}}$$

$$w_{c_{13}}=w_{c_{13A_{1}}}+w_{c_{13A_{2}}}+w_{c_{13A_{3}}}+w_{c_{13A_{4}}}+w_{c_{13A_{5}}}+w_{c_{13A_{6}}}+w_{c_{13A_{7}}}+w_{c_{13A_{8}}}+w_{c_{13A_{9}}}$$

$$+w_{c_{13A_{10}}}$$

$$w_{c_{12}}=w_{c_{12A_{1}}}+w_{c_{12A_{2}}}+w_{c_{12A_{3}}}+w_{c_{12A_{4}}}+w_{c_{12A_{5}}}+w_{c_{12A_{6}}}+w_{c_{12A_{7}}}+w_{c_{12A_{8}}}+w_{c_{12A_{9}}}$$

$$+w_{c_{12A_{10}}}$$

$$w_{c_{4}}=w_{c_{4A_{1}}}+w_{c_{4A_{2}}}+w_{c_{4A_{3}}}+w_{c_{4A_{4}}}+w_{c_{4A_{5}}}+w_{c_{4A_{6}}}+w_{c_{4A_{7}}}+w_{c_{4A_{8}}}+w_{c_{4A_{9}}}+w_{c_{4A_{10}}}$$

$$w_{c_{7}}=w_{c_{7A1}}+w_{c_{7A_{2}}}+w_{c_{7A_{3}}}+w_{c_{7A_{4}}}+ w_{c_{7A_{5}}}+w_{c_{7A_{6}}}+w_{c_{7A_{7}}}+w_{c_{7A_{8}}}+w_{c_{7A_{9}}}+w_{c_{7A_{10}}}$$

$$w_{c_{9}}=w_{c_{9A_{1}}}+w_{c_{9A_{2}}}+w_{c_{9A_{3}}}+w_{c_{9A_{4}}}+w_{c_{9A_{5}}}+w_{c_{9A_{6}}}+w_{c_{9A_{7}}}+w_{c_{9A_{8}}}+w_{c_{9A_{9}}}+w_{c_{9A_{10}}}$$

$$WA_{1}=w_{c_{14A_{1}}}+w_{c_{10A_{1}}}+w_{c_{11A_{1}}}+w_{c_{8A_{1}}}+w_{c_{6A_{1}}}+ w_{c_{1A_{1}}}+w_{c_{3A_{1}}}+w_{c_{5A_{1}}}+w_{c_{15A_{1}}}+w_{c_{2A_{1}}}$$

$$+ w_{c_{13A_{1}}}+w_{c_{12A_{1}}}+w_{c_{4A_{1}}}+w_{c_{7A_{1}}}+w_{c_{9A_{1}}}$$

$$WA_{2}=w_{c_{14A_{2}}}+w_{c_{10A_{2}}}+w_{c_{11A_{2}}}+w_{c_{8A_{2}}}+w_{c_{6A_{2}}}+ w_{c_{1A_{2}}}+w_{c_{3A_{2}}}+w_{c_{5A_{2}}}+w_{c_{15A_{2}}}+w_{c_{2A_{2}}}$$

$$+ w_{c_{13A_{2}}}+w_{c_{12A_{2}}}+w_{c_{4A_{2}}}+w_{c_{7A_{2}}}+w_{c_{9A_{2}}}$$

$$WA_{3}=w_{c_{14A_{3}}}+w_{c_{10A_{3}}}+w_{c_{11A_{3}}}+w_{c_{8A_{3}}}+w_{c_{6A_{3}}}+ w_{c_{1A_{3}}}+w_{c_{3A_{3}}}+w_{c_{5A_{3}}}+w_{c_{15A_{3}}}+w_{c_{2A_{3}}}$$

$$+ w_{c_{13A_{3}}}+w_{c_{12A_{3}}}+w_{c_{4A_{3}}}+w_{c_{7A_{3}}}+w_{c_{9A_{3}}}$$

$$WA_{4}=w_{c_{14A_{4}}}+w_{c_{10A_{4}}}+w_{c_{11A_{4}}}+w_{c_{8A_{4}}}+w_{c_{6A_{4}}}+ w_{c_{1A_{4}}}+w_{c_{3A_{4}}}+w_{c_{5A_{4}}}+w_{c_{15A_{4}}}+w_{c_{2A_{4}}}$$

$$+ w_{c_{13A_{4}}}+w_{c_{12A_{4}}}+w_{c_{4A_{4}}}+w_{c_{7A_{4}}}+w_{c_{9A_{4}}}$$

$$WA_{5}=w_{c_{14A_{5}}}+w_{c_{10A_{5}}}+w_{c_{11A_{5}}}+w_{c_{8A_{5}}}+w_{c_{6A_{5}}}+ w_{c_{1A_{5}}}+w_{c_{3A_{5}}}+w_{c_{5A_{5}}}+w_{c_{15A_{5}}}+w_{c_{2A_{5}}}$$

$$+ w_{c_{13A_{5}}}+w_{c_{12A_{5}}}+w_{c_{4A_{5}}}+w_{c_{7A_{5}}}+w_{c_{9A_{5}}}$$

$$WA_{6}=w_{c_{14A_{6}}}+w_{c_{10A_{6}}}+w_{c_{11A_{6}}}+w_{c_{8A_{6}}}+w_{c_{6A_{6}}}+ w_{c_{1A_{6}}}+w_{c_{3A_{6}}}+w_{c_{5A_{6}}}+w_{c_{15A_{6}}}+w_{c_{2A_{6}}}$$

$$+ w_{c_{13A_{6}}}+w_{c_{12A_{6}}}+w_{c_{4A_{6}}}+w_{c_{7A_{6}}}+w_{c_{9A_{6}}}$$

$$WA_{7}=w_{c_{14A_{7}}}+w_{c_{10A_{7}}}+w_{c_{11A_{7}}}+w_{c_{8A_{7}}}+w_{c_{6A_{7}}}+ w_{c_{1A_{7}}}+w_{c_{3A_{7}}}+w_{c_{5A_{7}}}+w_{c_{15A_{7}}}+w_{c_{2A_{7}}}$$

$$+ w_{c_{13A_{7}}}+w_{c_{12A_{7}}}+w_{c_{4A_{7}}}+w_{c_{7A_{7}}}+w_{c_{9A_{7}}}$$

$$WA_{8}=w_{c_{14A_{8}}}+w_{c_{10A_{8}}}+w_{c_{11A_{8}}}+w_{c_{8A_{8}}}+w_{c_{6A_{8}}}+ w_{c_{1A_{8}}}+w_{c_{3A_{8}}}+w_{c_{5A_{8}}}+w_{c_{15A_{8}}}+w_{c_{2A_{8}}}$$

$$+ w_{c_{13A_{8}}}+w_{c_{12A_{8}}}+w_{c_{4A_{8}}}+w_{c_{7A_{8}}}+w_{c_{9A_{8}}}$$

$$WA_{9}=w_{c_{14A_{9}}}+w_{c_{10A_{9}}}+w_{c_{11A_{9}}}+w_{c_{8A_{9}}}+w_{c_{6A_{9}}}+ w_{c_{1A_{9}}}+w_{c_{3A_{9}}}+w_{c_{5A_{9}}}+w_{c_{15A_{9}}}+w_{c_{2A_{9}}}$$

$$+ w_{c_{13A_{9}}}+w_{c_{12A_{9}}}+w_{c_{4A_{9}}}+w_{c_{7A_{9}}}+w_{c_{9A_{9}}}$$

$$WA_{10}=w_{c_{14A_{10}}}+w_{c_{10A_{10}}}+w_{c_{11A_{10}}}+w_{c_{8A_{10}}}+w_{c_{6A_{10}}}+ w_{c_{1A_{10}}}+w_{c_{3A_{10}}}+w_{c_{5A_{10}}}+w_{c_{15A_{10}}}$$

$$+w_{c_{2A_{10}}}+ w_{c_{13A_{10}}}+w_{c_{12A_{10}}}+w_{c_{4A_{10}}}+w_{c_{7A_{10}}}+w_{c_{9A_{10}}}$$
